# A distinct dimer configuration of a diatom Get3 forming a tetrameric complex with its tail-anchored membrane cargo

**DOI:** 10.1186/s12915-024-01933-x

**Published:** 2024-06-13

**Authors:** Chi-Chih Chen, Yu-Ru Huang, Yuen Ting Chan, Hung-Yun Lin, Han-Jia Lin, Chwan-Deng Hsiao, Tzu-Ping Ko, Tai-Wen Lin, Ya-Hsuan Lan, Hsuan-Ya Lin, Hsin-Yang Chang

**Affiliations:** 1 Department of Life Sciences and Institute of Genome Sciences, National Yang Ming Chiao Tung University, Beitou Dist, No. 155, Sec. 2, Linong St, Taipei City, 112304 Taiwan; 2 Department of Marine Biotechnology and Resources, National Sun Yat-Sen University, Kaohsiung, Taiwan; 3 Center of Excellence for the Oceans, National Taiwan Ocean University, Keelung, Taiwan; 4 Department of Bioscience and Biotechnology, National Taiwan Ocean University, Keelung City, Taiwan; 5 Institute of Molecular Biology, Academia Sinica, Taipei, Taiwan; 6 Institute of Biological Chemistry, Academia Sinica, Taipei, Taiwan

**Keywords:** Tail-anchored membrane protein, Posttranslational pathway, Get3, TRC40, ArsA

## Abstract

**Background:**

Most tail-anchored (TA) membrane proteins are delivered to the endoplasmic reticulum through a conserved posttranslational pathway. Although core mechanisms underlying the targeting and insertion of TA proteins are well established in eukaryotes, their role in mediating TA protein biogenesis in plants remains unclear. We reported the crystal structures of algal arsenite transporter 1 (ArsA1), which possesses an approximately 80-kDa monomeric architecture and carries chloroplast-localized TA proteins. However, the mechanistic basis of ArsA2, a Get3 (guided entry of TA proteins 3) homolog in plants, for TA recognition remains unknown.

**Results:**

Here, for the first time, we present the crystal structures of the diatom Pt-Get3a that forms a distinct ellipsoid-shaped tetramer in the open (nucleotide-bound) state through crystal packing. Pulldown assay results revealed that only tetrameric Pt-Get3a can bind to TA proteins. The lack of the conserved zinc-coordination CXXC motif in Pt-Get3a potentially leads to the spontaneous formation of a distinct parallelogram-shaped dimeric conformation in solution, suggesting a new dimer state for subsequent tetramerization upon TA targeting. Pt-Get3a nonspecifically binds to different subsets of TA substrates due to the lower hydrophobicity of its α-helical subdomain, which is implicated in TA recognition.

**Conclusions:**

Our study provides new insights into the mechanisms underlying TA protein shielding by tetrameric Get3 during targeting to the diatom’s cell membrane.

**Supplementary Information:**

The online version contains supplementary material available at 10.1186/s12915-024-01933-x.

## Background

A functionally diverse set of tail-anchored (TA) membrane proteins are delivered and inserted into the ER through a posttranslational pathway, termed the guided entry of TA protein (GET) system [1–4]. In eukaryotes, ranging from microbes to animals, the GET system is mediated by a cytosolic ATPase (termed as TRC40 or ASNA-1 in humans and Get3 in fungi), which coordinates the translocation of TA proteins to the ER by binding with ER-bound receptors (WRB/CAML in mammals, Get1/2 in yeast and *Arabidopsis*) [1, 2, 5–12].

Studies have extensively investigated the core machinery of TA proteins that is delivered into the ER membrane through the GET pathway, including the structures of protein complexes and genetic complementation [1, 2, 6, 8, 13–20]. Several solved crystal structures of yeast Get3 and its complex with Get1/2 or Get4/Get5 have been reported almost in the same time frame [6, 10, 21–24]. All these Get3 structures exist in open and nucleotide-bound closed (or semi-closed) dimeric states, coordinated through a zinc-bound CXXC motif. Subsequently, Mateja et al. examined a series of crystal structures of a TA-bound Get3 homodimer and identified a functional targeting complex for TA insertion [13]. McDowell et al. evaluated the cryo-electron microscopy (EM) structure of the human GET system complex, indicating that WRB/CAML insertase forms a heterotetramer for dimeric TRC40 recognition and TA insertion into the ER membrane [18]. Keszei et al. examined the cryo-EM structure of the metazoan pretargeting complex and demonstrated that Get4, Ubl4a (the mammalian homolog of Get5), Bag6, and SGTA (the mammalian homolog of Sgt2) act as a bridge for accurately loading TA proteins onto the Get3 dimer (from *Danio rerio*) [19]. However, the major discrepancy regarding the oligomeric state of either Get3 alone or the Get3–TA complex under physiological conditions remains obscure. Size-exclusion chromatography (SEC) findings have revealed that soluble Get3–TA complexes predominantly formed a tetramer, enabling Get3 to completely shield the transmembrane domain (TMD) from the aqueous environment [16, 17, 23, 25, 26]. However, to date, the crystal structure of only one tetrameric conformation (ADP-bound closed form) of the archaeal Get3 homolog has been reported [17].

Because various TA proteins are delivered to the ER, the chloroplast or mitochondrial outer membrane can be modulated by independent pathways; trafficking is more complex in plants than in other eukaryotic species [27–32]. Xing et al. identified two Get3 clades by performing phylogenetic analysis; they suggested the presence of alternative TA trafficking pathways in plants [33]. More than two Get3 paralogs are translated in plants, including *Chlamydomonas* (Cr-ArsA1 and Cr-ArsA2) [27], *Arabidopsis* (*At*Get3a, *At*Get3b, and *At*Get3c) [33], and *Phaeodactylum* (Pt-Get3a and Pt-Get3b in this work), whereas only one is present in eukaryotic microbes and animals. Plus, multiple TA isoforms have been found in plants; some of them are localized to the ER, whereas others are localized in the mitochondria and chloroplasts [34]. However, mechanisms through which the targeting factor distinguishes among these diverse trafficking signals remain to be elucidated.

Since the generation of the entire genome of the diatom *Phaeodactylum tricornutum* in 2008, phycological and genetic engineering studies have extensively employed it for the development of molecular tools and techniques and determined the function of every gene in diatom species [35]. In this study, we identified two isoforms of Get3 in *Phaeodactylum tricornutum*, a cytosolic Pt-Get3a and a chloroplast-localized Pt-Get3b protein. Furthermore, we solved the crystal structure of Pt-Get3a and determined that it does not contain the conserved CXXC motif, potentially leading to the spontaneous formation of a distinct parallelogram-shaped dimer in solution. Two parallelogram-shaped dimers are assembled in crystal packing to form an ellipsoid-shaped tetramer. This finding is consistent with our SAXS and biochemical results, indicating that the tetramerization of Get3 is necessary for TA protein targeting. The findings of mutational analysis also indicated the involvement of the helices α5 and α7 and the following TRC40-insert lid of Pt-Get3a in TA protein recognition.

## Results

### Overall structure of Pt-Get3a

The full-length Pt-Get3a (amino acids 1–349) was purified after its heterologous expression in *Escherichia coli*. The findings of size-exclusion chromatography (SEC) revealed that after the cleavage of the N-terminal 6His-tag by TEV protease, Pt-Get3a was predominantly present in the form of a tetramer under the near physiological saline condition (~ 100 mM NaCl). When the NaCl concentration was increased to 500 mM, the analysis of approximately 78 mL of the eluted volume revealed a single sharp peak corresponding to dimeric Pt-Get3a, indicating the effects of electrostatic interaction. These fractions were collected for crystallization (Additional file 1: Fig. S1) and ATPase activity assays (Additional file 1: Table S1). Pt-Get3a was successfully crystallized in the presence of nucleotide (ADP) at a resolution of 2.3 Å in the space group as C2221. Moreover, we observed the apo-form crystal of Pt-Get3a at a resolution of 3.8 Å in the space group as I2_1_2_1_2_1_ (Table 1.).

**Table 1.** Data collection and refinement statistics of Pt-Get3a

	**Nucleotide-free**	**ADP•AlF4**^**-**^
**Data Collection**		
Diffraction source	BL13B, NSRRC	BL13B, NSRRC
Space group	I2_1_2_1_2_1_	C222_1_
Cell dimensions		
a, b, c (Å)	107.3, 115.5, 151.8	101.3, 125.2, 120.6
α,β,γ (⁰)	90, 90, 90	90, 90, 90
Resolution (Å)	30-3.81 (3.95-3.81)	30-2.32 (2.40-2.32)
Wavelength (Å )	1.0000	1.0000
No. of observed reflections	66345	161989
No. of unique reflections	9530 (931)	32898 (3227)
R_merge_	0.076 (1.016)	0.037 (0.644)
I/σ(I)	24.1 (2.0)	38.7 (2.1)
Completeness (%)	99.9 (99.9)	98.0 (98.0)
Redundancy	7.0 (6.6)	4.9 (4.7)
**Refinement**		
Resolution (Å)	29.82-3.81 (3.94-3.81)	24.70-2.32 (2.40-2.32)
No. reflections	9017 (528)	31822 (2368)
R_work_/R_free_ (%)	32.0/36.9	19.3/24.4
R.m.s.d., bond lengths (Å)/ angles (°)	0.0018/0.46	0.0039/0.78
Average B factor (Å^2^)/No. of atoms		
Protein	36.9/5106	46.1/5092
Ligand		42.1/56
Water		50.5/632
Ramachandran plot, residues in (%)		
Favored	96.28	96.12
Allowed	3.72	3.88
Outliers	0	0
PDB code	8HAD	8HAC

**Fig. 1:**
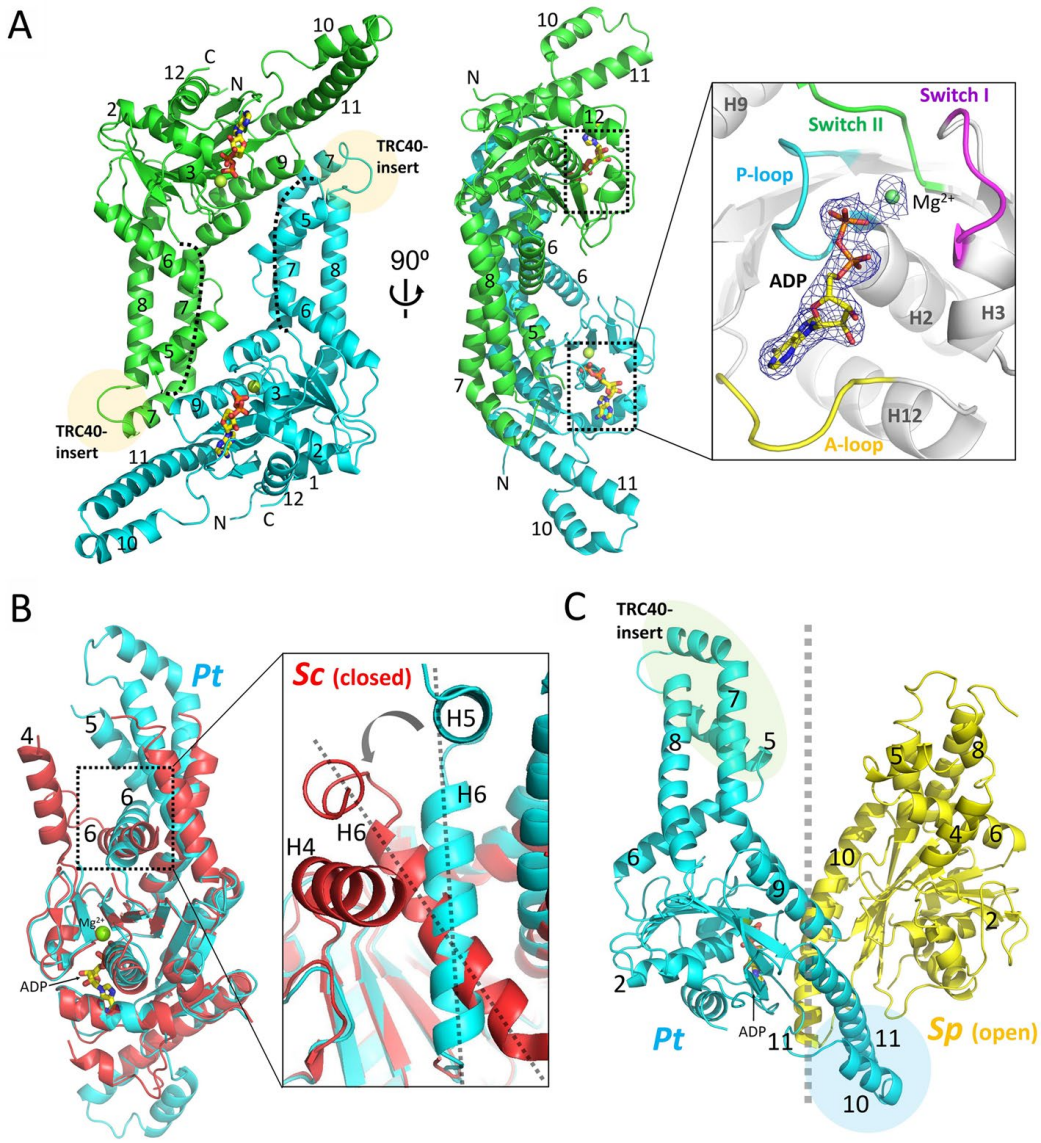
Crystal structure of an ADP-bound Pt-Get3a at a resolution of approximately 2.3 Å in the parallelogram-shaped dimer state. **A** Cartoon representation of the dimeric form of Pt-Get3a is shown in two orientations. The left monomer is colored in green for α-helices and β-strands, respectively, whereas the right monomer is in cyan. The disordered region connecting from β3 to α5 (including helix α4) in the α-helical subdomain is depicted by dotted lines. Semitransparent orange circles indicate the location of the TRC40-insert in the structure. The right panel presents Mg^2+^/ADP bound in the NBD and the surrounding P- (cyan) and A- (yellow) loops and switch I (magenta) and II (green) motifs are involved in nucleotide binding and catalysis. The Mg^2+^ ion and ADP are depicted by blue 2Fo-Fc electron density meshes (1.0 σ). **B** The monomer of Pt-Get3a (cyan) and the closed *Sc*Get3 (red, PDB: 2WOJ) are aligned in accordance with their ATPase domains. The helix α6 of Pt-Get3a located at the bottom of the α-helical subdomain moves inward movement compared with that of *Sc*Get3 in the closed state. **C** A split-view comparison of the monomer of Pt-Get3a (cyan) and *Sp*Get3 (yellow, PDB: 2WOO) in the open state. The semitransparent cyan circle indicates the helix-bundle motif, whereas the semitransparent green oval indicates extended and more ordered helices (α5, 7, and 8) and the TRC40-insert in the Pt-Get3a structure

**Fig. 2:**
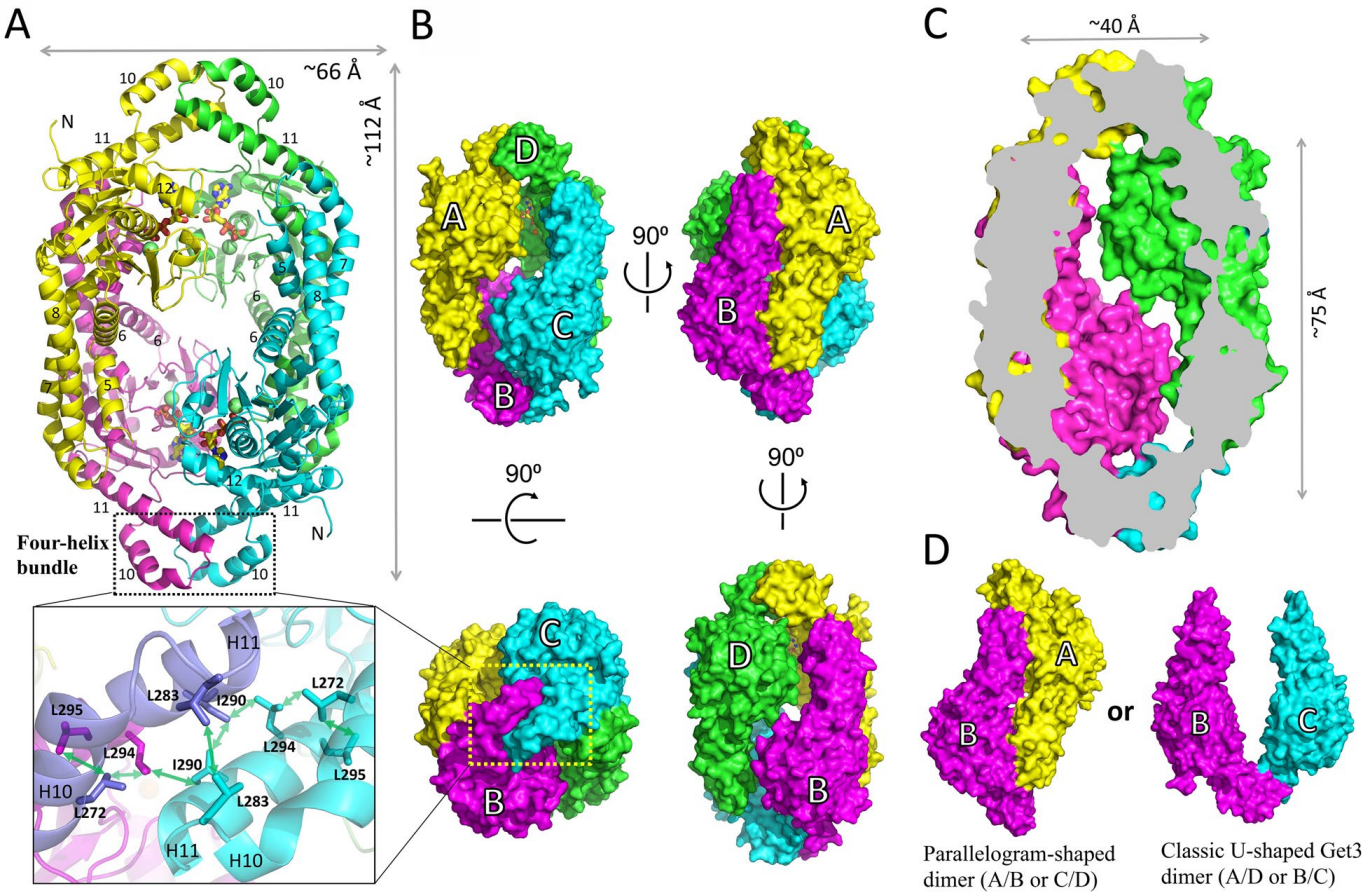
The ellipsoid-shaped Pt-Get3a tetramer. **A** Both nucleotide-free (I2_1_2_1_2_1_) and ADP-bound (C222_1_) structures contain four monomers assembled in two tetramers aligned along the long axis of symmetry in crystal packing. **B** Surface representation of the Pt-Get3a tetramer is shown in four orientations. Two parallelogram-shaped Pt-Get3a dimers [A (yellow)/B (magenta) and C (cyan)/D (green)] are assembled to form an ellipsoid-shaped tetramer. The bottom left panel depicts the molecular structure of the dimeric four-helix bundle in antiparallel orientation with hydrophobic interactions.** C** Surface representation of the central cavity of the ellipsoid-shaped Pt-Get3a tetramer cut through the middle. **D** Two crystallographic homodimers composed of either A/B or C/D (left) and A/D or B/C (right) monomers in crystal packing

Two molecules of Pt-Get3a in one asymmetric unit formed a homodimer in both apo and nucleotide-bound Pt-Get3a structures. In the nucleotide-bound structure (~ 2.3 Å), an ADP and a magnesium ion were observed in the two crystallographic independent monomers (Fig. 1; Additional file 1: Fig. S2). However, both the structures were similar, with their RMSD ranging from 0.47 to 0.87 Å over all backbone Cα atoms. Moreover, Pt-Get3a existed as a distinct parallelogram-shaped dimer in the crystal, which markedly differed from the structures of classic U-shaped Get3 dimers. The parallelogram-shaped dimer might form due to the lack of the zinc-bound CXXC motif, which coordinates the open-to-closed transition of Get3 for TA protein targeting. In addition, an ellipsoid-shaped tetramer of Pt-Get3a was observed through crystal packing in both crystal structures. Two parallelogram-shaped dimers (A/B and C/D) were assembled in a crystallographic tetramer aligned along the long axis of symmetry. The overall length and width of the tetrameric Pt-Get3a were approximately 112 Å and 66 Å, respectively (Fig. 2A and B). The size of the internal cavity of tetrameric Pt-Get3a was approximately 40 Å and 75 Å across the middle and down the long axis, respectively (Fig. 2C). The ellipsoid-shaped tetramer of Pt-Get3a could alternatively consist of two crystallographic homodimers of A/D and B/C monomers aligned along the short axis of symmetry (Fig. 2D), similar to the classic U-shaped formation of Get3 in the dimer state. Unless otherwise stated, we used the nucleotide-bound Pt-Get3a structure as a model for all figures.

**Fig. 3:**
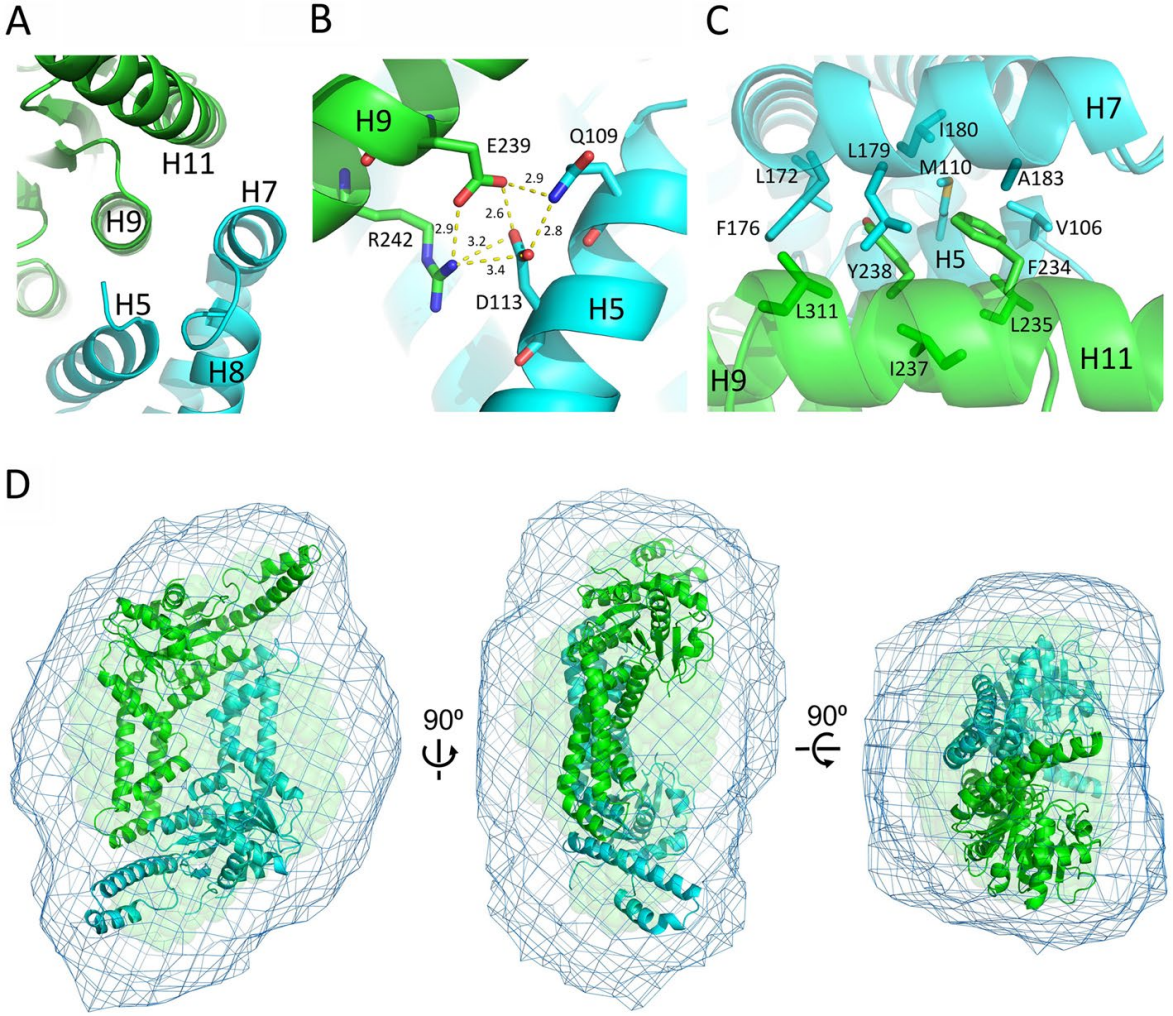
Parallelogram-shaped Pt-Get3 dimer in solution. **A** The molecular structure of the dimer interface A/B (or C/D) stabilized by four flanking helices through hydrophobic interactions, including α5 and α7 on one subunit (cyan) and α9 and α11 on the opposing subunit (green). **B** Two strictly conserved residues Glu239 and Arg242 of helix α9 form interchain salt bridges with Gln109 and Asp113 of helix α5 from the opposing monomer. **C** Hydrophobic interactions are formed in the extensive dimer interface. **D** SAXS structure of the parallelogram-shaped Pt-Get3a dimer (A/B or C/D) in solution. The averaged molecular SAXS envelope is shown in cyan mesh superposed on the Pt-Get3a dimer of the crystal structure by using the same color code as shown in Fig. 1A. The semitransparent green spheres represent the filtered envelope calculated by DAMFILT, which removes low occupancy and poorly connected atoms from the averaged envelope

Each monomer of Pt-Get3a possessed a core ATPase domain and an α-helical subdomain, similar to those observed in known Get3 proteins (Fig. 1). The nucleotide-binding domain of Pt-Get3a comprised a P-loop (β1-α2), switch I (β2-α3), switch II (β4-α7), and A-loop (β7-α12), which closely aligns with that of the yeast *Sc*Get3 structure (PDB ID: 2WOJ; Fig. 1B). The α-helical subdomain is formed by the helix α6 located at the base of the subdomain and additional helices (α5, 7α, and 8α) that lie above helix α6. Each monomer exhibits an open conformation, similar to the transition state of yeast *Sp*Get3 in the open form (PDB ID: 2WOO [14]; Fig. 1C). These regions, connecting helices α4-α5 and α7-α8 (including the TRC40-insert), might serve as a lid to protect the TMD of TA proteins from the aqueous cytosolic environment before their insertion into the ER [13, 14, 16, 36]. However, electron density in these regions is usually disordered or becomes more flexible than that in the rest of the protein in other Get3 structures. In our Pt-Get3a crystal structure, although the backbone density of approximately 14 residues connecting β3 to α5 (including helix α4) was missing, helices α5, 7α, 8α, and TRC40-insert were extended and more ordered in this parallelogram-shaped dimer conformation. This finding might be due to its complex residue–residue contact that forms stable hydrophobic and hydrophilic interactions between the dimer interface of A/B (or C/D), with a surface area of approximately 1750 Å^2^ in the crystal structure (calculated using PISA) [37]. We found that the two conserved residues Glu239 and Arg242 of helix α9 formed interchain salt bridges with the Gln109 and Asp113 of helix α5 from the opposing monomer (Fig. 3A and B). However, the double mutation E239A_R242A appeared to exert no effect on TA protein binding compared with its wild type (Additional file 1: Fig. S3). This phenomenon might be due to the fact that the extensive dimer interface is stabilized by a hydrophobic core formed by helices α9 (Phe234, Leu235, Ile237, and Tyr238) and α11 (Leu311 and Tyr312) on one monomer and helices α5 (Val106 and Met110) and α7 (Leu172, Phe176, Leu179, Ile180, and Ala183) from the opposing monomer (Fig. 3A and C). These hydrophobic residues on the helices involved in dimeric interactions are highly conserved among Get3 homologs (Additional file 1: Fig. S4). Our finding is consistent with that of previous mutagenesis experiments performed in archaeal *Mj*Get3 [17], suggesting that the hydrophobic residues Phe192 and Met196 of helix α8 (the equivalent residues being Leu179 and Ala183 in Pt-Get3a) are critical for the stabilization of the interface of the three-helix bundle in the tetramer structure.

**Fig. 4:**
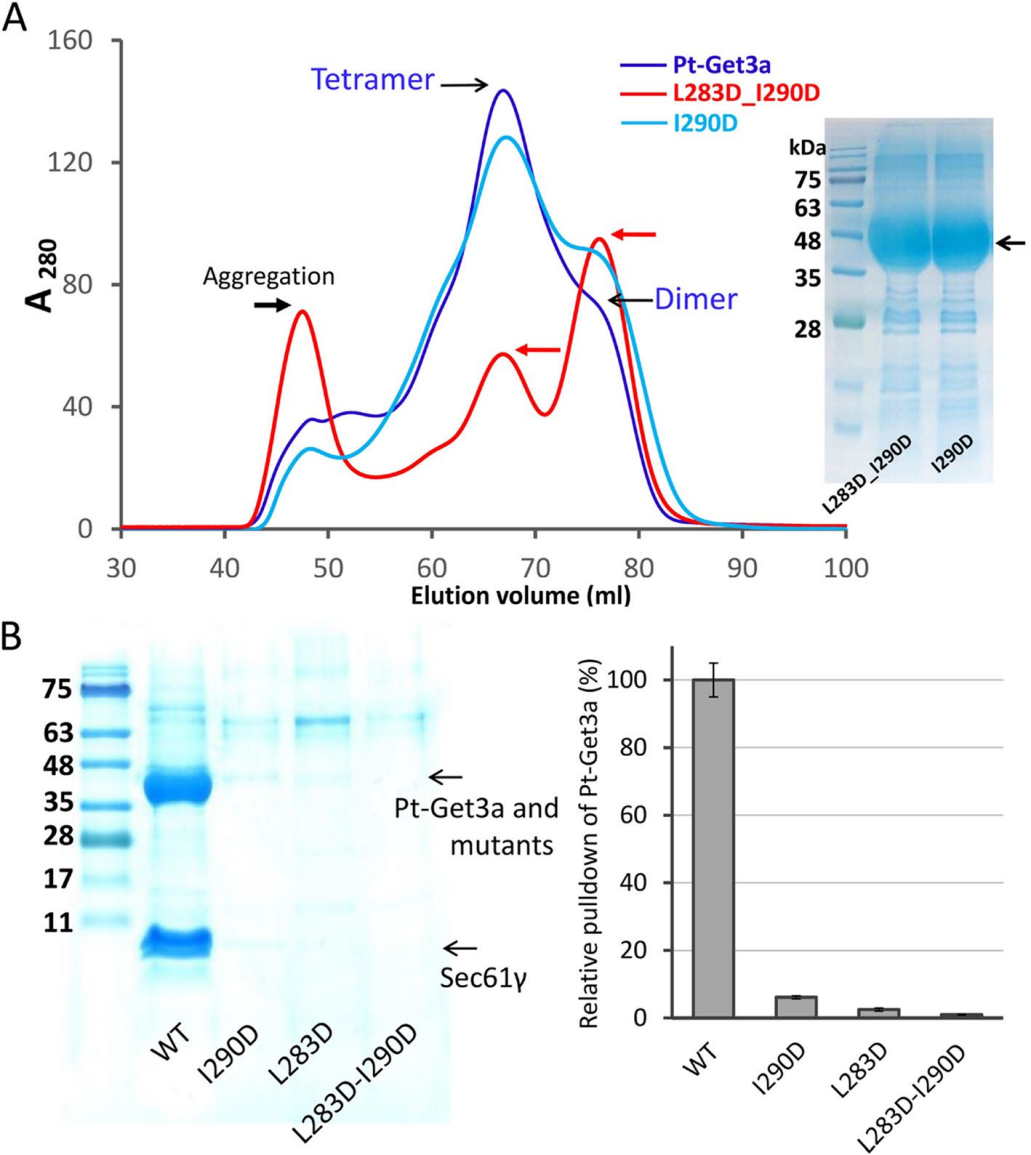
The four-helix bundle plays an essential role in tetramerization and TA binding. **A** SEC analysis of Pt-Get3a and its mutants, including the wild type (navy), I290D (light blue), and double mutation L283D_I290D (red) in the presence of approximately 100 mM NaCl buffer. The right SDS-PAGE image depicts the overexpression of purified Pt-Get3a mutants. **B** SDS-PAGE analysis of pulldown assay variants. Pt-Get3a and mutants were each purified using C-terminal 6 × His-tag-fused Sec61γ TA substrate through recombinant coexpression. The bar graph represents the quantitative analysis of the pulldown assay, and error bars denote the standard error of the mean. The copurified Pt-Get3a proteins were separated on ~ 10–12% TRIS-tricine gels and quantified using GelAnalyzer software (http://www.gelanalyzer.com). Each value is the average of three independent pulldown measurements

The crystal structure of Pt-Get3a may contain crystallographic homodimers (A/D and B/C) as an alternative (Fig. 2D). This dimer conformation is similar to those of classic Get3 structures, generally forming a U-shaped homodimer by the zinc-coordinated CXXC motif [14, 16, 20]. Because of the lack of the conserved CXXC motif, monomers in a U-shaped Pt-Get3a dimer (A/D or B/C) are associated through two α-helical hairpins (α10 and N terminus of α11) from each monomer, which are located at the equivalent area of the CXXC motif in other Get3 crystal structures. These two helical hairpin motifs create the majority of the dimer interface of A/D or B/C through a dimeric four-helix bundle in antiparallel orientation (Fig. 2; Additional file 1: Fig. S5). The dimer interface is predominantly hydrophobic and mainly stabilized by interfacial residues Leu283, Ile290, and Leu294 from each chain (Fig. 2). However, the A/D or B/C dimer exhibits a relatively small surface area of approximately 720 Å^2^ (calculated using PISA) in the crystal structure and fewer interactions than those of the parallelogram-shaped dimers (~ 1750 Å^2^) [37]. Although the presence of a larger surface area and the involvement of more interface residues in dimerization might result in a more energetically favorable association of protein subunits in solution, the parallelogram-shaped dimer (A/B and C/D) might be a crystal packing artifact. Therefore, we performed the SAXS analysis. The purified dimeric Pt-Get3a exhibited a linear Guinier plot at low *q* values, and the pair distribution *P*(*r*) indicated the presence of a single globular unit with a *D*_max_ of 130 Å. In addition, the radii of gyration (*R*_g_) values (*R*_g_ Guinier/*R*_g_ GNOM: 32.26/32.9 Å) are consistent with the crystal asymmetric unit and packing (*R*_g_ theoretical: 31.66 Å; Table 2.; Additional file 1: Fig. S6); these findings strongly support the presence of the parallelogram-shaped Pt-Get3a dimer (A/B and C/D) in solution (Fig. 3D). However, this raises a question of whether the four-helix bundle results in the higher-order oligomerization of the ellipsoid-shaped Pt-Get3a tetramer. We therefore created a single mutation I290D and a double mutation L283D_I290D within the dimer interface of the four-helix bundle. The single mutation I290D revealed a partial shift in the tetramer–dimer equilibrium in SEC. By contrast, the L283D_I290D mutation directly disrupted the hydrophobic interface, and its elution fractions predominantly shifted to the dimer form in the presence of approximately 100 mM NaCl (Fig. 4A). Compared with the wild-type variant, these mutants suppressed the activity of TA substrate binding (Fig. 4B). These data suggest that the dimeric four-helix bundle plays a structural role as an adapter in the association between two parallelogram-shaped dimers (A/B and C/D) to form a tetramer.

**Table 2.** SAXS statistics

	**Pt-Get3a dimer**	**Pt-Get3a opentetramer**	**Pt-Get3a/TA closed tetramer**	MjGet3 closed tetramer*
Rg theoretical	31.66	35.05	-	44.2
Rg Guinier	32.26	-	46.5	47
Rg GNOM	32.9	-	47.6	47.5
Dmax	130	-	155	165

*MjGet3 crystal structure (PDB ID: 3UG6) [ 17]

### Localization of Pt-Get3a protein

**Fig. 5:**
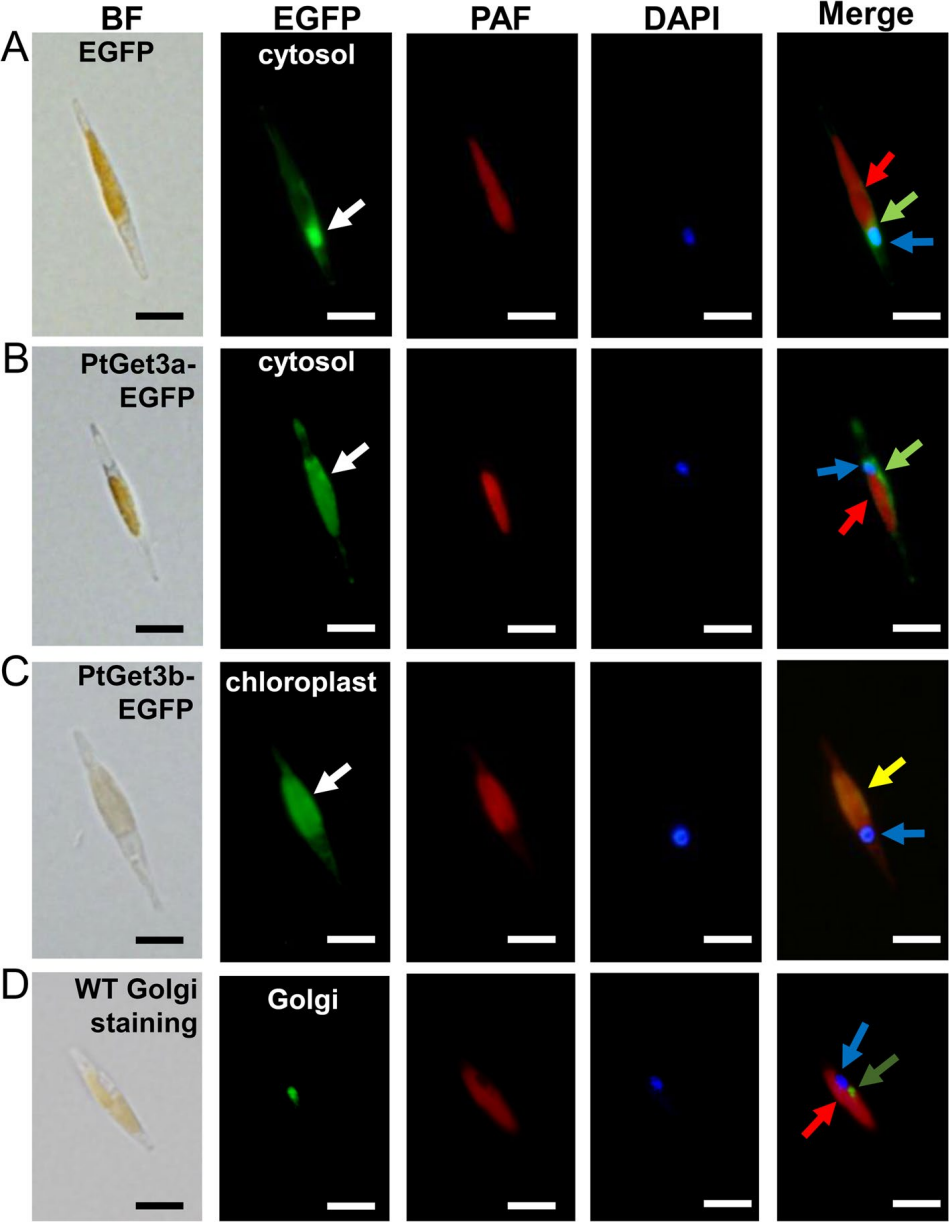
In vivo localization of EGFP fusion proteins in transgenic diatom strains through fluorescence microscopy. **A** The pNR-EGFP strain was used in a control group exhibiting the pattern of fluorescent proteins accumulated in the cytosol. **B** Pt-Get3a was detected in the cytosol. **C** Pt-Get3b was localized in the chloroplast. **D** The control group utilized wild-type *P. tricornutum* with Golgi and DAPI staining, displaying the pattern of plastid autofluorescence (red), the organelle localization of Golgi (dark green), and the nucleus (blue). BF, bright field; EGFP, EGFP fluorescence; PAF, plastid autofluorescence; DAPI, nucleus staining with DAPI dye; Merge, a merge of all fluorescence channel. In the EGFP channel, the white arrows represent the location of the EGFP or Pt-Get3-EGFP fusion proteins. In the Merge channel, the red, blue, and dark green arrows represent the location of the plastid, nucleus, and Golgi, respectively. The light green arrow represents the cytosolic location of the EGFP or Pt-Get3a-EGFP fusion protein, whereas the yellow arrow represents the colocalization of the plastid and fusion protein of Pt-Get3b-EGFP. The scale bars represent 10 µm

The alignment of the primary and secondary structures of Pt-Get3a with other homologs is presented in Fig. S4 (Additional file 1). Pt-Get3a exhibited approximately 50% sequence identity to the green alga *Chlamydomonas reinhardtii* Cr-ArsA2, suggesting that the diatom Get3 has a similar catalytic function to Cr-ArsA2. Plus, all critical residues in yeast *Sc*Get3 (Phe246, Tyr250, Glu253, Gln257, Glu258, Asp265, Tyr298, Glu304, Glu307, Asp308, and Glu320), which are required for binding to Get1, Get2, and Get4, are highly conserved in Pt-Get3a (Additional file 1: Fig. S7). Through the genome mining of the genus *Phaeodactylum*, the potential candidates of Sgt2 and Get4 homologs (sequence ID: XP_002176724.1 and XP_002178199.1) could be identified using BLASTP. These findings indicate the presence of a diatom GET system with a similar molecular mechanism of TA protein targeting as observed in yeast. Because two Get3 genes (*PtGet3a* and *PtGet3b*) are encoded in *P. tricornutum*, we identified the subcellular localization of two Pt-Get3 homologs. Two C-terminally EGFP-tagged fusions (Pt-Get3a-EGFP and Pt-Get3b-EGFP) were generated and transformed through multipulse electroporation (Additional file 1: Fig. S8; Table S2). Fluorescence microscopy analysis revealed two distinct patterns of subcellular localization. Pt-Get3a-EGFP was localized in the cytosol, whereas Pt-Get3b-EGFP, containing a predicted transit peptide of approximately first 80 amino acids, was detected in the chloroplast (Fig. 5). This result is consistent with our sequence alignment data suggesting that Pt-Get3a can transfer ER-destined TA proteins to cytosol-exposed membranes in diatom cells.

### Pt-Get3a pulldown assay for TA substrate binding

**Fig. 6:**
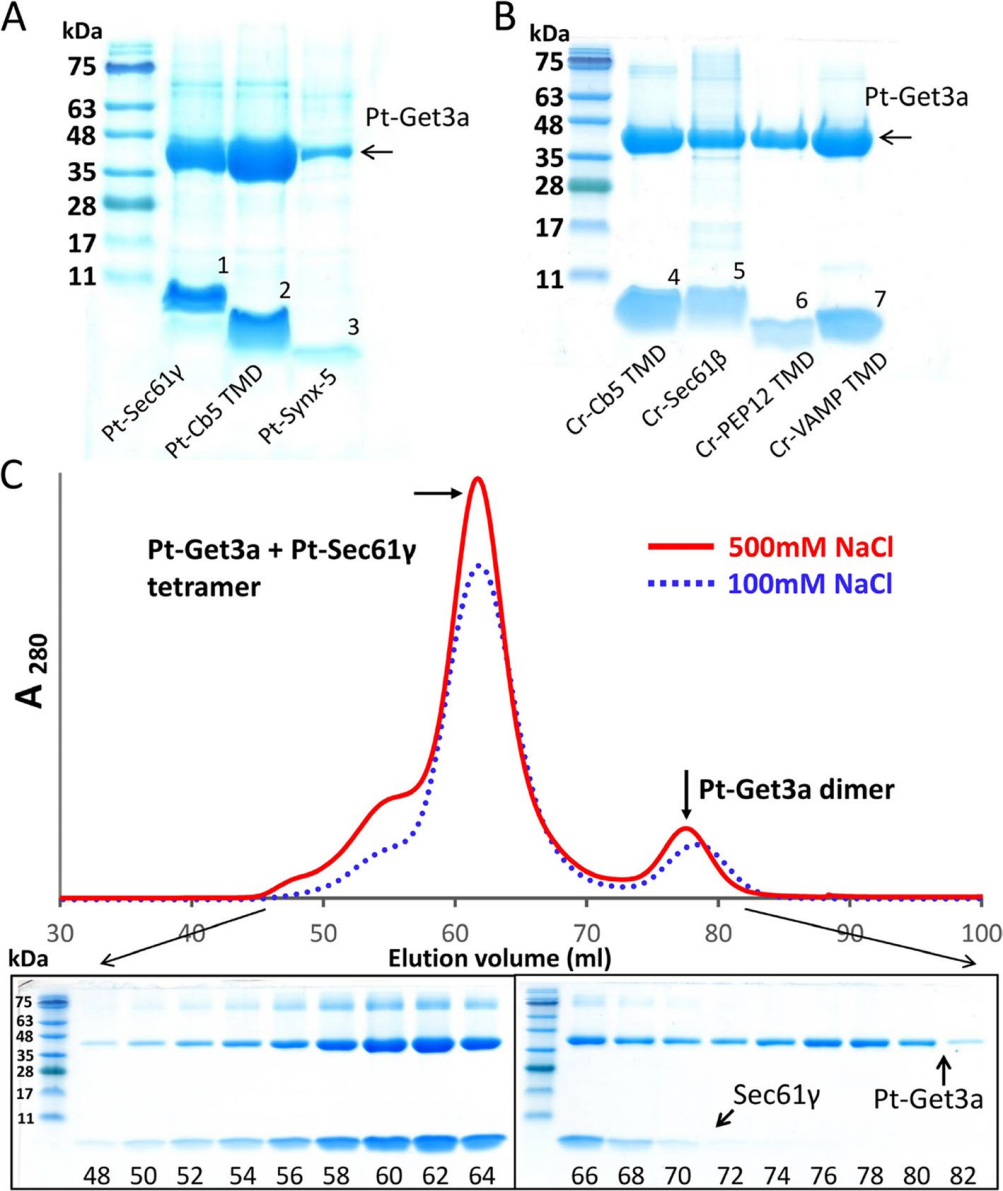
Pt-Get3a/TA complex in the tetrameric state. **A** Characterization of the Pt-Get3a protein pulldown assays for Pt-TA substrate. **B** Pulldown assays for Cr-TA substrate interactions. Arrows and numbers indicate Pt-Get3a/Sec61γ (1), Pt-Cb5-TMD (2), Pt-Synx5 (3), Cr-Cb5-TMD (4), Cr-Sec61β (5), Cr-PEP12-TMD (6), and Cr-VAMP-TMD (7). **C** SEC analysis of the Pt-Get3a/Sec61γ complex in the presence of approximately 100 mM (blue dotted line) and 500 mM NaCl (red line) by using the Superdex-200–16/600 GL column on an AKTA Purifier FPLC (GE Healthcare). The lower panel depicts the SDS-PAGE analysis of Pt-Get3a/Sec61γ complex purified through SEC in the presence of approximately 100 mM NaCl with Superdex-200–10/300 GL column

We examined the TA protein recognition activity of Pt-Get3a by performing coexpression and pulldown assays by using various C-terminal 6xHis-tag-fused TMDs of TA proteins [30, 31]. We found that the cytosolic Pt-Get3a can bind to certain ER or vesicle TA substrates from *P. tricornutum* and *C. reinhardtii* (Fig. 6A and B). However, Pt-Get3a can also bind to the TMD of cytochrome *b*5 (Cb5 TMD), which has been proposed to be independent of GET system [38]*.* Unless otherwise stated, we used Pt-Sec61γ as a model target substrate.

To determine whether TA proteins are appropriately shielded by the oligomeric state of Pt-Get3a, we performed SEC to identify the approximate size of the targeting complex under the physiological saline condition (100 mM NaCl). SEC findings revealed that the recombinant Pt-Get3a/Sec61γ complex predominantly exists as a tetramer in solution (Fig. 6C; Additional file 1: Fig. S9). To rule out the possibility of electrostatic interactions, we conducted a control experiment in which we increased the NaCl concentration to 500 mM. The results indicated no significant disruption of the Pt–Get3a/TA tetramer or a shift in the tetramer-dimer equilibria, indicating that the formation of a stable tetramer is required for TA protein binding (Fig. 6C; Additional file 1: Fig. S9). By contrast, a small fraction (~ 78 mL of eluted volume) was purified as dimeric Pt-Get3a without TA protein binding. However, previous studies have reported that TA proteins can be delivered to either dimeric or tetrameric Get3 to form a functional targeting complex. Thus, we performed SEC to determine the size of the recombinant Get3/TA complex obtained from either yeast or zebra fish. We observed that a small pool of the Get3 tetramer (or oligomer) bound to TA proteins could always convert into the Get3/TA dimer but not degrade to dimeric Get3 alone in SEC (Additional file 1: Fig. S10). These results suggest that only the tetrameric Pt-Get3a can bind to TA proteins. This hypothesis is supported by the findings of mutagenesis and pulldown assays (Fig. 4; Additional file 1: Fig. S11), in which the mutants I290D and L283D_I290D tend to form a predominant dimer, presumably adopting a parallelogram-shaped structure (A/B and C/D), which results in a significant loss of their capability to bind to the Sec61γ substrate (Fig. 4B). Together, these findings indicate that the efficiency of TA binding is cooperatively dependent on the tetramerization of Pt-Get3a.

### In-solution analysis of the Pt-Get3a/TA complex

To obtain the molecular architecture of Pt-Get3a in the TA-bound tetramer state, we performed SAXS analysis by using the purified Pt-Get3a/TA complex. The analysis of approximately 62 mL of the eluted volume revealed a single sharp peak corresponding to a tetrameric Pt-Get3a/Pt-Cb5-TMD complex in solution (Additional file 1: Fig. S12). The resultant curve of the TA-bound Get3 complex exhibited a linear Guinier plot at low *q* values, and the pair distribution *P* indicated the presence of a single globular unit with a *D*_max_ of 155 Å (Additional file 1: Fig. S13A and B). The radii of the gyration (*R*_g_) values of the closed Pt-Get3a/TA tetramer (*R*_g_ Guinier/*R*_g_ GNOM: 46.5/47.6 Å) differed from the crystal structure of ellipsoid-shaped Pt-Get3a in the open tetramer state (*R*_g_ theoretical: 35.05 Å), suggesting an open-to-closed conformational change in the Pt-Get3a tetramer upon TA binding (Table 2.; Additional file 1: Fig. S13C). The generated molecular envelope of the Pt-Get3a/TA complex is similar to the dimensions of *Mj*Get3 in the closed tetramer state (*R*_g_ Guinier/*R*_g_ GNOM: 47/47.5 Å) [17], indicating that the conformation of the Pt-Get3a/TA tetramer is similar to that of the closed *Mj*Get3 tetramer (Table 2.; Additional file 1: Fig. S13D).

### Selectivity of Pt-Get3a interaction with TA substrate

**Fig. 7:**
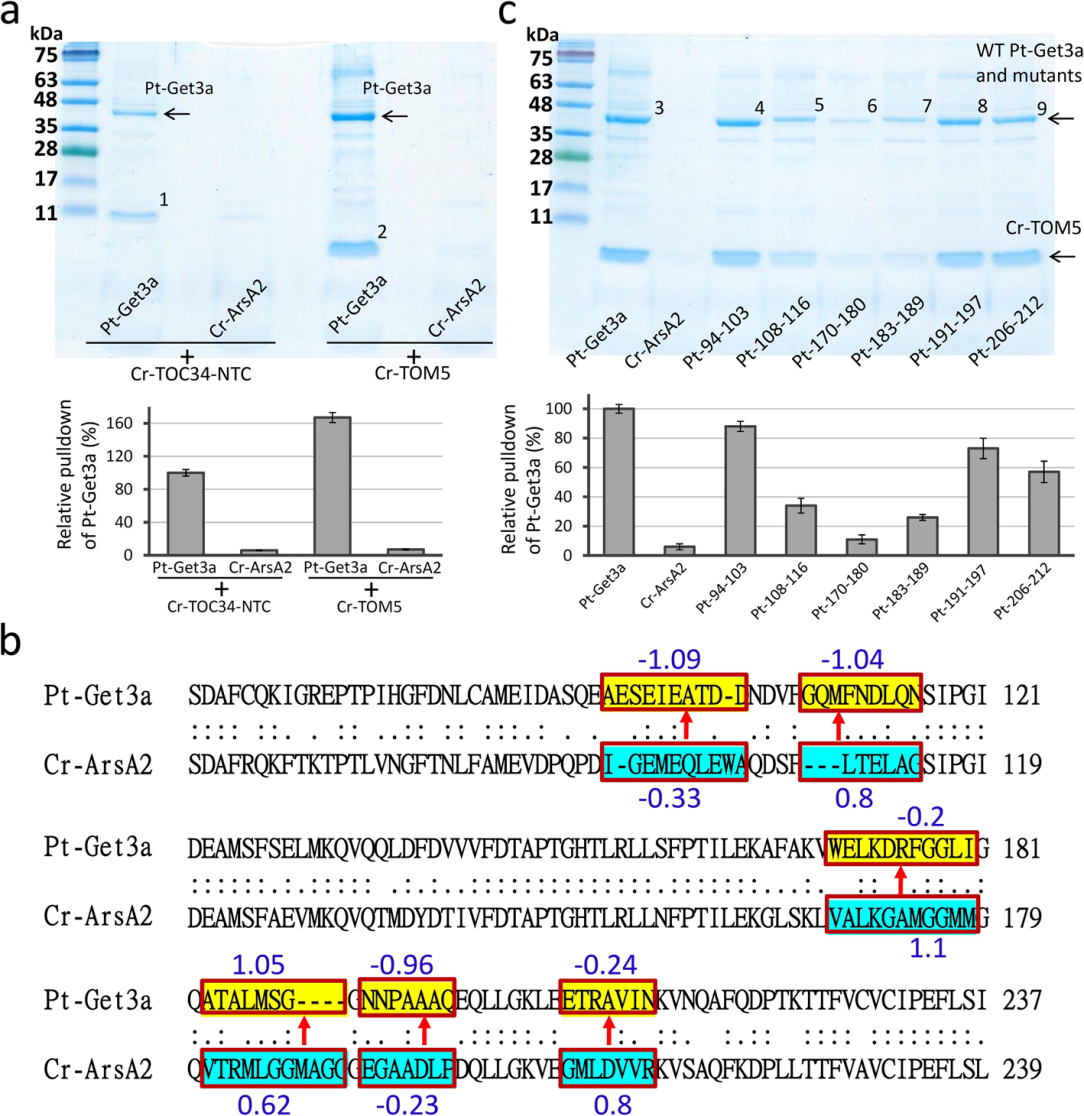
Selectivity of Pt-Get3a protein interaction with chloroplast and mitochondrial TA proteins. **A** Pt-Get3a proteins were each purified with C-terminal 6 × His-tag fused TA substrates through recombinant coexpression. Arrows and numbers indicate Pt-Get3a/Cr-TOC34-NTC (1) and Pt-Get3a/Cr-TOM5 (2). **B** Six chimeric fraction switch variants of Pt-Get3a (yellow boxes) were designed by the substitution of the equivalent region of Cr-ArsA2 (blue boxes). The blue numbers indicate the GRAVY scores of the fraction variants. **C** Characterization of six chimeric mutations, denoted as Pt_94-103 (3), Pt_108-116 (5), Pt_170-180 (6), Pt_183-189 (7), Pt_191-197 (8), and Pt_206-212 (9), pulldown assays compared with wild type (3) for Cr-TOM5 TA substrate interactions. The SDS-PAGE and bar graph represents the quantitative analysis of pulldown assay variants, as described in Fig. 4

To identify the members of chloroplast- or mitochondria-TA proteins in *P. tricornutum* and evaluate the selectivity of Pt-Get3a in TA recognition, we used several chloroplast or mitochondrial outer membrane protein (TOC or TOM) sequences of *C. reinhardtii* as baits with BLASTP. However, no potential candidates were found, probably due to low sequence identity and similarity between the target and template. Thus, we examined two previously constructed green alga TA substrates [31], Cr-TOC34 NTC (including the TMD and its upstream N-terminal 10 amino acids and downstream C-terminal sequence regions) and Cr-TOM5, by conducting an in vitro test. Pt-Get3a exhibited significant binding activity for both chloroplast- and mitochondria-TA substrates. As a negative control, Cr-ArsA2 did not interact (or very little) with them (Fig. 7A). This finding suggests that Pt-Get3a exhibits less specific selectivity to interact with TA proteins.

Pt-Get3a has more than 50% sequence identity of amino acids to Cr-ArsA2 but exhibits distinct features in TA discrimination. On the basis of this discrepancy, we conducted a comparative experiment between Pt-Get3a and Cr-ArsA2 to determine how targeting factors distinguish TA proteins. Six chimeric fraction switch variants of Pt-Get3a, denoted as Pt_94-103, Pt_108-116, Pt_170-180, Pt_183-189, Pt_191-197, and Pt_206-212, respectively, were created by the substitution of the equivalent region of Cr-ArsA2 after the alignment of the amino acid sequences of Pt-Get3a and Cr-ArsA2 (Fig. 7B). Chimeric mutations were generated on the basis of two criteria as follows: (1) the equivalent regions had a high ratio of different amino acids in a given position and (2) regions of Pt-Get3a must include conserved TA-binding residues located in the helices α4-α5, α7-α8, and TRC40-insert of the α-helical subdomain. Because mitochondria-TA exhibits a higher binding affinity to Pt-Get3a, we used Cr-TOM5 as a bait to capture those chimeric mutations, which we anticipated would result in the loss of TA protein binding capability, thereby disrupting interactions with Pt-Get3a. The pulldown assay revealed that Pt_108-116, Pt_170-180, and Pt_183-189 lost their binding activity for the Cr-TOM5 substrate (approximately 3- to tenfold decrease) compared with their wild type (Fig. 7C). According to our Pt-Get3a structure, all the three regions are located at the top of α-helical subdomain (α5, α7, and TRC40-insert) and closely interact with each other. These regions are equivalent to the helices α5 and α7-α8 of *Mj*Get3, which form a hydrophobic chamber for shielding TA proteins in the closed tetramer model, supporting the importance of these three regions for TA recognition and targeting in Pt-Get3a (Additional file 1: Fig. S14). It is noteworthy that two of these regions exhibited lower hydrophobicity (− 1.04 in 108–116 fragments and − 0.2 in 170–180 fragments) than did the equivalent regions in Cr-ArsA2 (0.8 in 109–114 fragments and 1.1 in 168–178 fragments; Fig. 7B). The lower hydrophobicity of the α-helical subdomain of Pt-Get3a may result in the nonspecific binding of TA substrates, such as Cr-TOM5 and Cr-TOC34 TMD, with relatively lower TMD hydrophobicity (Additional file 1: Table S3) [39–41].

## Discussion

Many studies have proposed that the conserved zinc-coordinated CXXC motif is essential for Get3 to act as a hinge point for the dimerization and open-to-closed transition during TA chaperoning. However, Suloway et al. determined that an archaeal Get3 (*Tk*Get3) from *Thermococcus kodakarensis* does not contain the CXXC sequence but can bind to TA proteins in the oligomer state [17]. We recently characterized the green algal Cr-ArsA1 monomer and Cr-ArsA2 oligomer and determined that both of them lack the CXXC motif and could bind to certain TA substrates [30, 31]. Primary and tertiary structure analyses revealed that Cr-ArsA1 is a pseudodimer that encodes two homologous domains connected to each other through two extended helices α12 and α12′ (equivalent to α11 in *Sc*Get3 and *Mj*Get3) in the domain swapped–like structure, which may functionally replace the zinc-coordinated CXXC motif of classic Get3 proteins [31]. In the current study, we noted that because of the lack of the CXXC motif, the U-shaped dimer (A/D or B/C) in crystal packing folds through a dimeric four-helix bundle in antiparallel orientation via a hydrophobic interaction. Since only a small surface area is buried in this region, the interface of the U-shaped dimer could not be stable as observed in the classic Get3 dimer or Cr-ArsA1. This hypothesis was supported by the finding of SAXS analysis, which indicated that the parallelogram-shaped Pt-Get3a dimer (A/B and C/D) was present.

We observed that Pt-Get3a predominantly existed as a tetramer under the physiological saline condition (~ 100 mM NaCl) in SEC. However, because of electrostatic interactions, the tetramer complex was not stable in solution, as indicated by the appearance of a dimer peak in SEC when the salt concentration was increased. By contrast, such disruption of a tetramer to a dimer was not observed for the Pt-Get3a-TA complex even in the presence of a high salt concentration. Moreover, the pulldown assays of a series of Pt-Get3a mutants followed by SEC revealed positive cooperativity toward TA binding and Pt-Get3a tetramerization. These results indicate that the formation of a stable and functional tetramer is required for TA targeting. This finding was further confirmed by the results of SAXS and biochemical analyses, which indicated the presence of a TA-bound Pt-Get3a tetramer in solution. Our data are consistent with those of previous quantitative mechanistic analyses, which reported that efficient TA binding requires the transient formation of a Get3 tetramer [25]. Therefore, we determined that the four-helix bundle structure of Pt-Get3a plays a structural role as an adapter for the association between two parallelogram-shaped dimers (A/B and C/D) to form a tetramer during TA protein binding. This distinct molecular machinery could be a general feature in other Get3 proteins lacking the CXXC motif, such as algal Cr-ArsA2, archaeal *Tk*Get3, and *Arabidopsis At*Get3a.

In the GET pathway in yeast, the pretargeting factor Sgt2 initially forms a complex with the TA protein prior to delivering it to the ATP-bound Get3. Several structural and biochemical experiments revealed that a homodimer Get3 interacts with two copies of the Get4–Get5 complex during TA transfer from Sgt2 to Get3. However, the exact protein composition of the complex of Get3 or TA-bound Get3 under physiological conditions has been a matter of debate over the past decade, mainly owing to its tetramer or oligomer composition being the predominant one in solution [16, 25, 42]. A breakthrough was achieved when Suloway et al. proposed that the crystal structure of archaeal *Mj*Get3 forms an elongated dumbbell-shaped tetramer in the closed ADP-bound state, although no TA substrate was bound in the structure [17]. In this work, we observed that the Pt-Get3a structure forms a distinct ellipsoid-shaped tetramer in the open ADP-bound state, which markedly differs from the closed *Mj*Get3 tetramer in the spatial arrangement. This difference can be due to complex residue–residue contact in the extensive dimer interface of A/B and C/D in open Pt-Get3a than that in closed *Mj*Get3. In the *Mj*Get3 model, interactions forming the closed tetramer undergo primarily hydrophobic packing through the three-helix bundle composed of helices α4, α5, and α8 from different subunits across the tetramer. Unlike *Mj*Get3, our Pt-Get3a tetramer model revealed that each subunit underwent a large conformational change into an open state, mainly stabilized by four flanking helices through hydrophobic interactions, including α5/α7 (equivalent to α5/α8 of *Mj*Get3) on one subunit and α9/α11 (equivalent to α10/α11 of *Mj*Get3) from the opposing subunit in the crystal structure (Fig. 3). Numerous hydrophobic residues on those helices are conserved among Get3 homologs, and some of them are even crucial for TA protein binding [13, 14, 20, 31] (Additional file 1: Fig. S4). Another two strictly conserved residues Glu239 and Arg242 of helix α9 may play a crucial structural role in the stabilization of the open tetramer by forming interchain salt bridges with the conserved Asp113 of helix α5 in the Pt-Get3a structure (Fig. 3B). Such a difference in conformational arrangement results in the formation of a large and relatively hydrophilic internal cavity in the Pt-Get3a crystal structure, which may represent the initiating mode of the open tetramer prior to TA protein binding in the cytosol. It is noteworthy that the equivalent residues of Glu239 and Arg242 in Pt-Get3a are Glu251 and Arg254 in yeast *Sc*Get3 or Glu253 and Arg256 in *Mj*Get3 (Additional file 1: Fig. S4), which generally form interchain hydrogen bonds with other two conserved residues (Asn61 and Asp64 in yeast *Sc*Get3 and Ser67 and Asp70 in *Mj*Get3) in closed form structures, presumably stabilizing the extensive dimer interface into a more compact dimeric conformation for TA protein binding (Additional file 1: Figs. S4 and S15). These two equivalent residues in Pt-Get3a are also conserved (Asn61 and Asp64) and likely form interchain hydrogen bonds with Glu239 and Arg242, thus resulting in the formation of a closed tetramer (Additional file 1: Fig. S15). Similarly, the residue Asp113 of Pt-Get3a is highly conserved, being either an aspartate or glutamate in the equivalent location in other Get3 homologs (Asp128 in *Sc*Get3, Glu126 in *Mj*Get3, and Glu134 in human ASNA1; Additional file 1: Fig. S4). These findings indicate that modulation required to change the composition of Get3 complexes is likely mediated by a cluster of evolutionarily conserved networks of amino acids, thus possibly resulting in the formation of a tetrameric conformation in most Get3 homologs.

The C-terminal region of TA proteins (including the TMD) contains organelle-specific targeting signals that direct them into appropriate subcellular locations. Those molecular signals may include multiple physicochemical properties, such as hydrophobicity, helical propensity, charge distribution, and amino acid variations in its C-terminal region [43–45]. The highly hydrophobic TMD directs TA proteins to the ER through the yeast GET pathway [46]. In this study, by analyzing various chimeric mutations between Pt-Get3a and Cr-ArsA2, we determined that not only the features of the TMD but also the hydrophobicity of the TA binding groove of Get3 could contribute to TA protein targeting. Our data revealed that the lower hydrophobicity of the α-helical subdomain of Pt-Get3a may lead to the nonspecific binding of TA substrates localized in the chloroplast or mitochondria (generally having lower TMD hydrophobicity) and thus may increase the risk of the mislocalization of TA proteins in diatoms.

## Conclusions

**Fig. 8:**
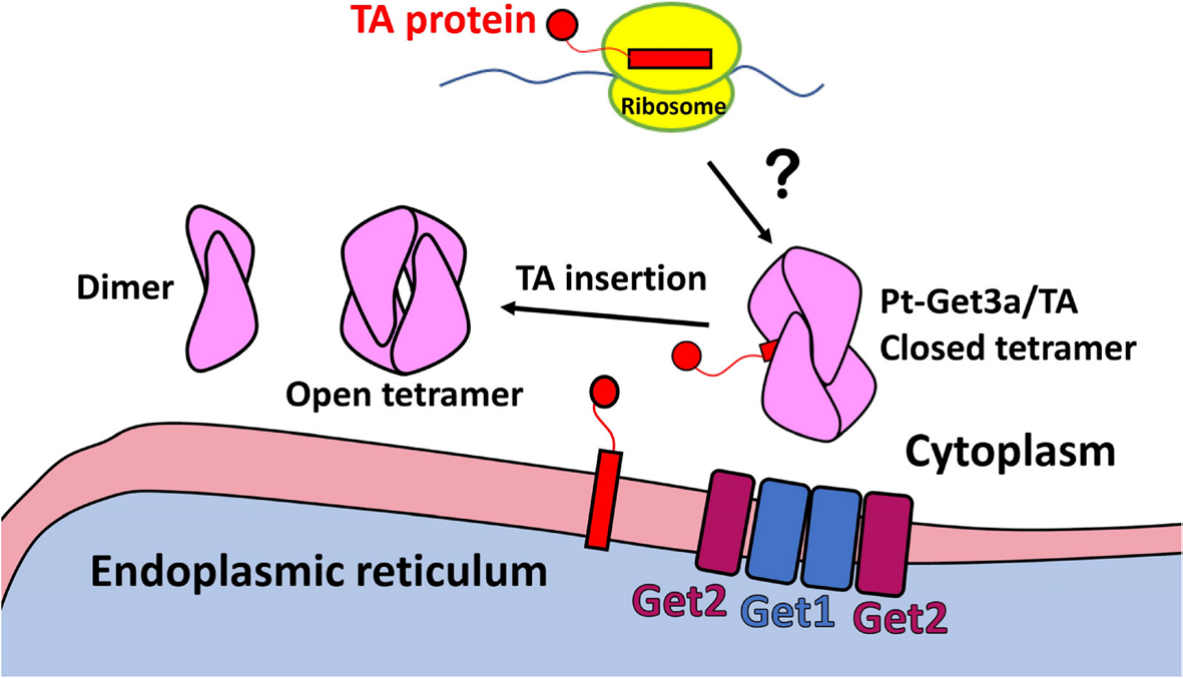
Model for Get3/TA complex. A physically plausible role of Pt-Get3a in posttranslational membrane targeting in the diatom *P. tricornutum*. Question mark (?): unknown pre-targeting factor(s) could be involved

Our structural and functional data suggest that the tetrameric Get3 model acts as a TA transport cage, protecting its TMD at the hydrophobic chamber from the cytosolic environment during trafficking to the membrane. The soluble Pt-Get3a likely exists in equilibrium between a dimer with a parallelogram shape and a tetramer with an open ellipsoid shape in the cytosol. These findings may enable us to build a physically plausible model of posttranslational TA protein targeting by Pt-Get3a in the diatom *P. tricornutum* (Fig. 8). This study provides insights into mechanisms underlying TA protein shielding by the diatom Get3 tetramer during targeting to the membrane. Our findings can be applicable to other plant species.

## Methods

### Protein crystallization and X-ray data collection

The purification of Get3 and Get3-TA complex for biochemical assays was performed as previously described [31]. For the crystallization of the cleaved His-tag free recombinant protein, we obtained crystals using full-length Pt-Get3a. The native crystals obtained from initial conditions were further refined manually. Finally, for apo form Pt-Get3a, we selected the crystallization condition of 0.07 M sodium acetate, pH4.6, 5.6% PEG4000, and 30% glycerol. The crystals were grown via the sitting-drop vapor-diffusion method at 25 °C by mixing the Pt-Get3a (~ 20 mg/mL) with an equal volume of a reservoir solution. Crystallization of nucleotide-bound (ADP-AlF_4_^−^) Pt-Get3a was selected: 0.085 M sodium acetate, pH 4.7, 0.25 M ammonium acetate, 18.5% glycerol, 0.25 M sodium bromide, with 24.5% polyethylene glycol (PEG) 4000. The crystals were grown via the sitting-drop vapor-diffusion method at 25 °C by mixing the Pt-Get3a (~ 5 mg/mL) solution containing 2 mM ADP, 2 mM MgCl_2_, 2 mM AlCl_3_, and 8 mM NaF with an equal volume of crystallization buffer. X-ray data were collected at beamlines TLS-13C and -15A of the National Synchrotron Radiation Research Center (Hsinchu, Taiwan). All diffraction data were processed and scaled with the HKL-2000 package [47] and detailed statistics are presented in Table 1..

### Structure determination and refinement

The crystal structure of nucleotide-bound Pt-Get3a was solved using the molecular replacement method, with the program MOLREP and using the structure of yeast Get3 (Protein Data Bank ID: 2WOJ) as the search model [14]. The solved structure then served as a starting model for apo form Pt-Get3a crystal. Initial model rebuilding and structural modifications were performed using COOT [48]. The resulting model was subjected to computational refinement with the program REFMAC5. Several rounds of model adjustment with COOT and refinement with PHENIX were performed using 2.32 Å (nucleotide-bound) and 3.81 Å (apo) resolution datasets to improve the quality and completeness of the structure [49]. The final refinement statistics are listed in Table 1..

### Small-angle X-ray scattering

The small-angle X-ray scattering was performed at the beamline (BL23A1), National Synchrotron Radiation Research Center (NSRRC), Hsinchu, Taiwan. The purified recombinant proteins were concentrated to about 5 mg/mL. For SAXS data collection, the purified Pt-Get3a (0.1 mL) was injected into the HPLC system equipped with an online size exclusion column at a flow rate of 0.035 mL/min. The experimental parameters were as follows: photon energy, 15 keV; distance-to-sample, 4 m. For the dimeric form, the scattering vector (*q*) ranged from 0.00043 to 0.247 Å^–1^, and from 0.00048 to 0.151 Å^–1^ for the tetrameric Pt-ArsA2/TA protein complex, where *q* = 4πsinθ/λ, 2θ = the scattering angle, and λ = the wavelength of the X-ray.

The experimental scattering profiles were corrected for background scattering by the solvent, and then the particle distance distribution profiles were calculated using GNOM program [50]. Ten independent runs for dummy residue modeling for each sample were performed using the *GASBOR program* in the package ATSAS [51] online within a spherical search diameter of *D*_max_ = 130 Å with a symmetry constraint of P2 for dimer Pt-Get3a, and 155 Å with a symmetry constraints of p222 for Pt-Get3a/TA protein complex. These final models were aligned and averaged using *SUPCOMB* and *DAMSEL* in the *DAMAVER* package [52]. The filtered cut-off shapes were calculated by DAMFILT to remove low occupancy atoms with default setting.

### Pulldown assay

Pulldown assays were conducted as described previously with modifications [31]. *E. coli* BL21 (DE3) was co-transformed with the pET21 vector carrying expression cassette of wild type or mutated Get3 homolog, and with pET28 vector containing an expression cassette for a series of C-terminally His-tagged TA proteins. For TA substrates with high-molecular-mass proteins (MW > 10 kDa), such as Cb5, VAMP, PEP12, and TOC34, we used a N-terminally truncated fragment, containing the predicted TMD domain and the remaining C-terminal residues, for pull down assay (Additional file 1: Table S3). All these genes were synthesized by Genomics (Taipei, Taiwan). Sequence verification was performed by Mission Biotech (Taiwan).

For the experimental details, coexpression was carried out at 22 °C for ~ 20 h by induction with 0.1 mM IPTG after the cells reached an OD600 of ~ 0.5. Basically, 3 L of cell culture was routinely maintained per batch for each pulldown assay to purify targeting complexes as our previously reported protocol [31]. After cell disruption in the presence of protease inhibitors using sonication, the supernatant was batch purified by Ni–NTA affinity chromatography. The elution fractions were concentrated to a final volume of ~ 20 mL. For a standard analysis, 15 µL of various copurified Get3-TA complexes were analyzed by 12% Tris-tricine gels, and each Get3 protein band was quantified using GelAnalyzer software (GelAnalyzer 2010a by Istvan Lazar, www.gelanalyzer.com).

### ATPase activity assays

ATPase activity was determined by using a colorimetric ATPase assay kit (Innova Biosciences). The reaction mixture (200 µL) contained 10 mM Tris–HCl pH 7.5, 150 mM NaCl, 5 mM MgCl_2_, 1 mM ATP, and purified Pt-Get3a proteins (~ 20 µg). The reaction was incubated at 30 °C for 5 min and quenched by adding 50 µL of PiColorLock mix reagent (Expedeon). The released phosphate was measured at 600 nm and quantified based on the phosphate standard curve.

### Fluorescence microscopy of various transgenic diatom strains

The diatom *P. tricornutum* (strain CCMP 632) was obtained from the Provasoli-Guillard National Center for Marine Algae and Microbiota (East Boothbay, ME, USA) and maintained on sterilized f/2 medium at 20 °C with an irradiance level of 115 µmol photons/m^2^/s 24 h per day [53]. The gene sequence of *PtGet3a* (XP_002183697.1) and *PtGet3b* (XP_002178015.1) were amplified from genomic DNA of *P. tricornutum* by PCR, and they were respectively ligated to the intermediate sequence between nitrate reductase promoter (pNR) and reporter gene (*enhanced green fluorescence protein, EGFP*) by Gibson Assembly Cloning Kit (New England Biolabs, Ipswich, MA, USA) to yield pNR-Pt-Get3a-EGFP and pNR-Pt-Get3b-EGFP vectors. All PCR primers used in this study are listed in Table S4. All plasmids were expressed by *E. coli* DH5α stain, purified, and linearized by the *Ahd*I site, and then used in electroporation experiments.

All operating procedures refer to previous report and make minor adjustments [54]. *P. tricornutum* was transferred to EASW medium in advance to cultivate it to the logarithmic growth phase. The diatom cells were collected and washed twice with 375 mM sorbitol solution. Then, 2 × 10^8^ cells were mixed with 5 µg of linearized transgenic vector and 40 µg of salmon sperm DNA (Sigma-Aldrich, St. Louis, MO, USA). After an ice bath for 30 min, the sample was transferred to a 2-mm electroporation cuvette. Diatom electroporation used Model PA-4000 Advanced PluseAgile electrotransplantation system (Cyto Pulse Sciences Inc., Glen Burnie, MA, USA) to generate multiple series of square wave pulses, including 8 poring pulses and 5 transferring pulses (Additional file 1: Table S2). The diatoms that received multiple square wave pulses were immediately transferred to the EASW medium and cultured for 24 h. Subsequently, 5 × 10^7^ diatom cells were smeared on 5 EASW agar plates (1.5%) containing 100 µg/mL Zeocin. About 20 − 100 brownish colonies appeared under 2 − 3 weeks of incubation at 20 °C with continuous illuminated which were individually transferred into 2 mL EASW medium containing 100 µg/mL Zeocin. After the 7-day screening period, PCR and green fluorescence measurement were used to detect surviving cells to verify the presence and expression of the reporter gene (EGFP). Additionally, a fluorescent microscope (Optiphot-2, Nikon, Tokyo, Japan) was used to observe the transgenic diatoms under 1000 × magnification.

Extraction of the diatom genome was performed using the EasyPrep HY Genomic DNA Extraction Kit (Biotools, Taipei, Taiwan) and follow the manufacturer’s instructions. Each PCR reaction contains 1 ng of genomic DNA, 500 nM specific primer pairs, and 1 × Gran Turismo PreMix (Ten Giga Bio, Keelung, Taiwan). The primers used to detect *EGFP* and the reference gene *ribosomal protein small subunit 30S* (RPS) were listed in Table S4. The reaction was carried out in a thermal cycler (GeneAtlas G02, ASTEC, Fukuoka, Japan) and the PCR conditions were as follows: 10 min preheating at 95 °C; an amplification process with 32 cycles of 95 °C for 20 s, 60 °C for 20 s, and 72 °C for 30 s; a final cycle of 72 °C for 5 min, then holding at 14 °C.

### Fluorescent staining of diatom cells

After 4 days of cultivation in L1 medium, a mixture comprising 900 µL of diatom solution containing 10^6^ cells and 100 µL of 37% formaldehyde was employed to fix the cells. The fixed cells were then rinsed with L1 medium for twice. Subsequently, 1 mL of enhance solution (5 µM BSA, 10 mM HEPES, and 0.5 × HBSS), 2 µL of BODIPY™ FL C5 Golgi fluorescent dye (Invitrogen™, CA, USA), and 1 µL of DAPI dye (sigma) were added. After incubation of 90 min, the stained cells were resuspended in L1 medium to prepare slides for fluorescence microscope observation (Olympus BX61, OLYMPUS, Tokyo, Japan) at a magnification of 400 × .

### Supplementary Information

Additional file1: Figure S1 Purification and characterization of Pt-Get3a protein. Figure S2 Omit maps of bound nucleotides. Figure S3 SDS-PAGE analysis of pulldown assay. Figure S4 Multiple sequence alignment of Get3 homologs. Figure S5 Structure of the helix bundle motif. Figure S6 SAXS solution structural analysis of Pt-Get3a dimer. Figure S7 Conserved residues in yeast ScGet3 involved in Get1, Get2, and Get4 binding are highlighted in dark blue. Figure S8 Conformation of introduced fragments in transgenic diatom strains. Figure S9 SEC analysis of the Pt-Get3a/TA complex in the presence of approximately 100 mM NaCl. Figure S10 SEC and SDS-PAGE analysis of Get3/TA complex. Figure S11 SDS PAGE analysis of coexpression of Pt-Get3a and Sec61γ substrate. Figure S12 TA-bound Pt-Get3a complex in the tetrameric state. Figure S13 SAXS solution structural analysis of the Pt-Get3a/TA tetramer complex. Figure S14 The α-helical subdomain of the open Pt-Get3a tetramer and closed MjGet3 tetramer. Figure S15 Structural comparison. Table S1 ATPase activity assay. Table S2 Parameters of multipulse electroporation. Table S3 Sequences, properties, and functions of TA substrates examined in this study. Table S4 PCR primers used in this study.

## Data Availability

All data generated or analyzed during this study are included in this published article and its additional files. Coordinates and structure factors have been deposited in the RCSB Protein Data Bank with the accession codes 8HAD and 8HAC [55, 56].

## Abbreviations

TA: Tail-anchored
ArsA: Arsenite transporter
ER: Endoplasmic reticulum
Get3: Guided entry of TA proteins 3
TRC40: Transmembrane domain recognition complex subunit of 40 kDa
ASNA: Arsenical pump-driving ATPase protein
WRB: Tryptophan-rich basic protein
CAML: Calcium-modulating cyclophilin ligand
TMD: Transmembrane domain
EM: Electron microscope
SAXS: Small-angle X-ray scattering
SEC: Size-exclusion chromatography
TEV: Tobacco etch virus
PISA: Protein interfaces surfaces and assemblies
TOC: Chloroplast outer membrane protein
TOM: Mitochondrial outer membrane protein
NTC: TMD and its N- and C-terminal regions
