# Supplementary material for: A distinct dimer configuration of a diatom Get3 forming a tetrameric complex with its tail-anchored membrane cargo
Source: BMC Biol. 2024 Jun 13;22:136. doi: 10.1186/s12915-024-01933-x (PMC11170914; doi:10.1186/s12915-024-01933-x)
Supplement: Supplementary file 1 — Additional file1: Figure S1 Purification and characterization of Pt-Get3a protein. Figure S2 Omit maps of bound nucleotides. Figure S3 SDS-PAGE analysis of pulldown assay. Figure S4 Multiple sequence alignment of Get3 homologs. Figure S5 Structure of the helix bundle motif. Figure S6 SAXS solution structural analysis of Pt-Get3a dimer. Figure S7 Conserved residues in yeast ScGet3 involved in Get1, Get2, and Get4 binding are highlighted in dark blue. Figure S8 Conformation of introduced fragments in transgenic diatom strains. Figure S9 SEC analysis of the Pt-Get3a/TA complex in the presence of approximately 100 mM NaCl. Figure S10 SEC and SDS-PAGE analysis of Get3/TA complex. Figure S11 SDS PAGE analysis of coexpression of Pt-Get3a and Sec61γ substrate. Figure S12 TA-bound Pt-Get3a complex in the tetrameric state. Figure S13 SAXS solution structural analysis of the Pt-Get3a/TA tetramer complex. Figure S14 The α-helical subdomain of the open Pt-Get3a tetramer and closed MjGet3 tetramer. Figure S15 Structural comparison. Table S1 ATPase activity assay. Table S2 Parameters of multipulse electroporation. Table S3 Sequences, properties, and functions of TA substrates examined in this study. Table S4 PCR primers used in this study. [file 12915_2024_1933_MOESM1_ESM.docx]

**Additional Files**

**A distinct dimer configuration of a diatom Get3 forming a tetrameric complex with its tail-anchored membrane cargo**

Chi-Chih Chen^1,2,#^, Yu-Ru Huang^1,#^, Yuen Ting Chan^1^, Hung-Yun Lin^3^, Han-Jia Lin^4^, Chwan-Deng Hsiao^5^, Tzu-Ping Ko^6^ , Tai-Wen Lin^5^, Ya-Hsuan Lan^1^, Hsuan-Ya Lin^1^, Hsin-Yang Chang^1, #, *^

^1^ Department of Life Sciences and Institute of Genome Sciences, National Yang Ming Chiao Tung University, Taipei, Taiwan.

^2^ Department of Marine Biotechnology and Resources, National Sun Yat-sen University, Kaohsiung, Taiwan

^3^ Center of Excellence for the Oceans, National Taiwan Ocean University, Keelung, Taiwan

^4^ Department of Bioscience and Biotechnology, National Taiwan Ocean University, Keelung City, Taiwan

^5^ Institute of Molecular Biology, Academia Sinica, Taipei, Taiwan

^6^ Institute of Biological Chemistry, Academia Sinica, Taipei, Taiwan

**Running title:** A tetrameric Get3 for tail-anchor targeting

**Keywords:** tail-anchored membrane protein, post-translational pathway, GET3, TRC40, ArsA

**Corresponding author:**

Hsin-Yang Chang, Ph.D

Department of Life Sciences and Institute of Genome Sciences

National Yang Ming Chiao Tung University

No. 155, Sec. 2, Linong St. Beitou Dist., Taipei City 112304, Taiwan.

Phone: +886-2-2826-7168

E-mail: hychang5@nycu.edu.tw

# These authors contributed equally to this work.

**Table S1. ATPase activity assay.**

| ATPase | *V_max_* (nmols/min/mg) | relative *V_max_^a^* |
| --- | --- | --- |
| Cr-ArsA2*^b^* | 183.2 ± 6.2 | 1 |
| Cr-ArsA1*^b^* | 166.0 ± 4.4 | 0.91 |
| Pt-Get3a | 179.0 ± 9.3 | 0.98 |

*^a^ V_max_* relative to that of wild type Cr-ArsA2

*^b^* Kinetic data obtained from our previous publication (Lin et al., 2019)

**Table S2: Parameters of multi-pulse electroporation**

|  | Initial voltage | Pulse  time | Pulse interval | Voltage decay rate | Number of pulses |
| --- | --- | --- | --- | --- | --- |
| poring pulses | 300 V | 50 ms | 50 ms | 10% | 8 |
| transferring pulses | 8 V | 50 ms | 50 ms | 40% | 5 |

**Table S3:** **Sequences, properties, and functions of TA substrates examined in this study**

| TA-protein | Amino acid sequences of the full length or N-terminal truncated TA proteins used here (predicted TMD residues are indicated in bold and underlined) | GRAVY score of TMD^a^ | Function^b^ |
| --- | --- | --- | --- |
| Pt-Cb5^c^ | MSAEKEYILDEISQHTTTESCWLIIGNASNGGPKVYDVTKYLDDHPGGAEVMLDVAGQDADEFFEDIGHSKEARAELKNYLVGNFKIDAATLAKMKADAEAKAQQKNS**GTGIMLIVLMALFAIAYGYY**QTQMK | 1.77 | Heme/Steroid binding domain, electron transfer |
| Pt-Sec61γ^c^ | MSESKEGFKEVLIEPLQQFAKDSMHLVKKCTKPDRKEFTAIARATG**VGFL IMGFIGFFVKLIHIPIN**NILVGN | 1.82 | Protein translocation complex, gamma subunit of ER-translocon |
| Pt-Syntaxin5^c^ | …….RSNRQ**LMLRLFSVLVVFIIVFVIGF**A | 2.85 | Golgi-localized SNARE protein |
| Cr-Cb5 | …….TRTFH**VILPLLILALALALNFYMMS**QKKSA | 2.23 | Heme/Steroid binding domain, electron transfer |
| Cr-VAMP | …….CRMKL**IVLFAILLLAVVIFLLVCF**TGGKNCTK | 3.56 | Endosomal R-SNARE protein, Vamp7/Nyv1-family (R.III) |
| Cr-PEP12 | …….GRNKC**LLVALVAAAIVSVLLIIIL**T | 3.37 | Endosomal Qa-SNARE protein, Syntaxin7/Syx7/Pep12/Syp2-family |
| Cr-Sec61β | MAGSQSQASTLVARGGRSSSNAPTGAAGALRRRPVRGSGGSGTTKAQQAAMNFYTDDTPGWKMSP**VVVITMSLSFIAFVTILHVV**GKFQ | 2.47 | Protein translocation complex, beta subunit of ER-translocon |
| Cr-TOM5 | MASNDLLTSAQKWLREEYTNEAKFGANLRLLR**GIAIFGGAVFVFRNFGEALF**GA | 1.38 | One subunit of translocase of outer mitochondrial membrane complex, a large complex of the mitochondrial outer membrane that mediates transport of proteins into all mitochondrial compartments. |
| Cr-TOC34 | …….HPRLSSKPSHRFR**WLLPVAIAAEVLFYRRFL**HPRLDDNQRRVEREEERVWALRGQQRRALGLHRPHRPDKDAAWRLEQMYDDD | 1.27 | Translocon at the outer envelope membrane of chloroplasts, GTPase |

^a^ Grand average of hydropathy (GRAVY) value for protein sequences (http://www.gravy-calculator.de/)

^b^ Functional annotations for TA proteins are based on the phytozome (http://www.phytozome.net)

^c^ Indicating plasmids constructed in this study

**Table S4:** **PCR primers used in this study**

| Gene | Name | Sequence |
| --- | --- | --- |
| *Pt-Get3b* | Pt_Get3b-Fw | CGAACGgaattcGCCACCATGGTTATCGCGCTGC |
|  | Pt_Get3b-Rv | CTTGCTCACCATaccggtAATTTCACGCAGCAGCT |
| *Pt-Get3a* | Pt_Get3a-Fw | CGAACGgaattcGCCACCATGGACGACCTGGACC |
|  | Pt_Get3a-Rv | CTTGCTCACCATaccggtTTCATCCATATCAACCGGATC |
| *EGFP* | EGFP-Fw | TATAGAATTCATGGTGAGCAAGGGCGA |
|  | EGFP-Rv | CTTGTACAGCTCGTCCATG |
| *RPS* | RPS-Fw | CGAAGTCAACCAGGAAACCAA |
|  | RPS-Rv | GTGCAAGAGACCGGCATACC |


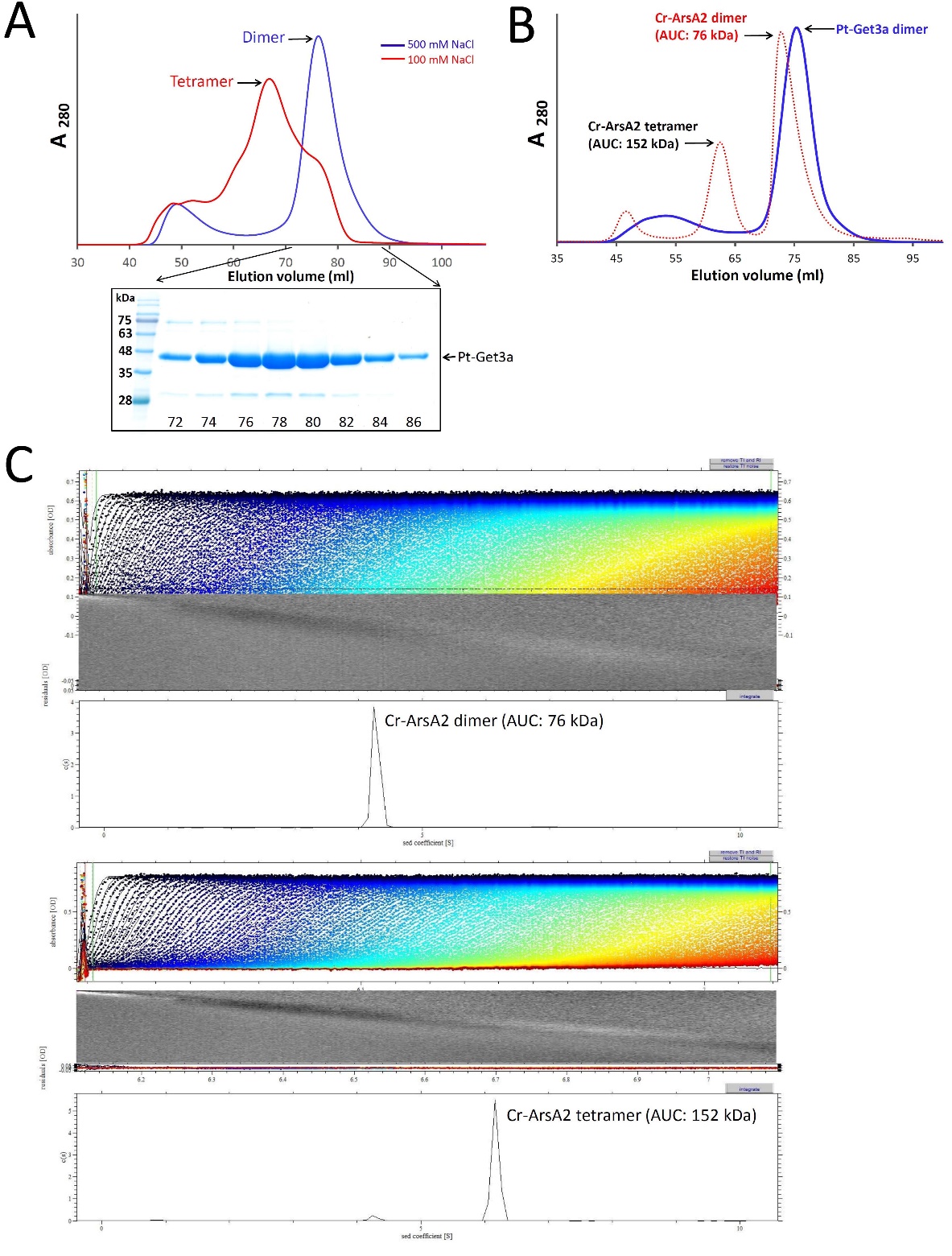


**Figure S1.** **Purification and characterization of Pt-Get3a protein.**

**A.** SEC analysis of purified recombinant Pt-Get3a in the presence of approximately 100 mM (red line) and 500 mM NaCl (blue line). The lower panel depicts the findings of the SDS-PAGE analysis of Pt-Get3a purified through SEC in the presence of approximately 500 mM NaCl. The elution fractions 72-86 were collected for crystallization and ATPase activity assays. **B.** Purified Pt-Get3a reveals a dimeric architecture in the presence of approximately 500 mM NaCl (blue line), with a molecular weight of approximately 70 kDa based on SEC performed using a Superdex-200-16/600 GL column on an AKTA Purifier FPLC (GE Healthcare). The red dotted line indicates a protein marker with a molecular weight of 76 kDa for dimeric Cr-ArsA2 and 152 kDa for tetrameric Cr-ArsA2 protein. **C.** Molecular weight determination of Cr-ArsA2 protein through analytical ultracentrifugation.

**
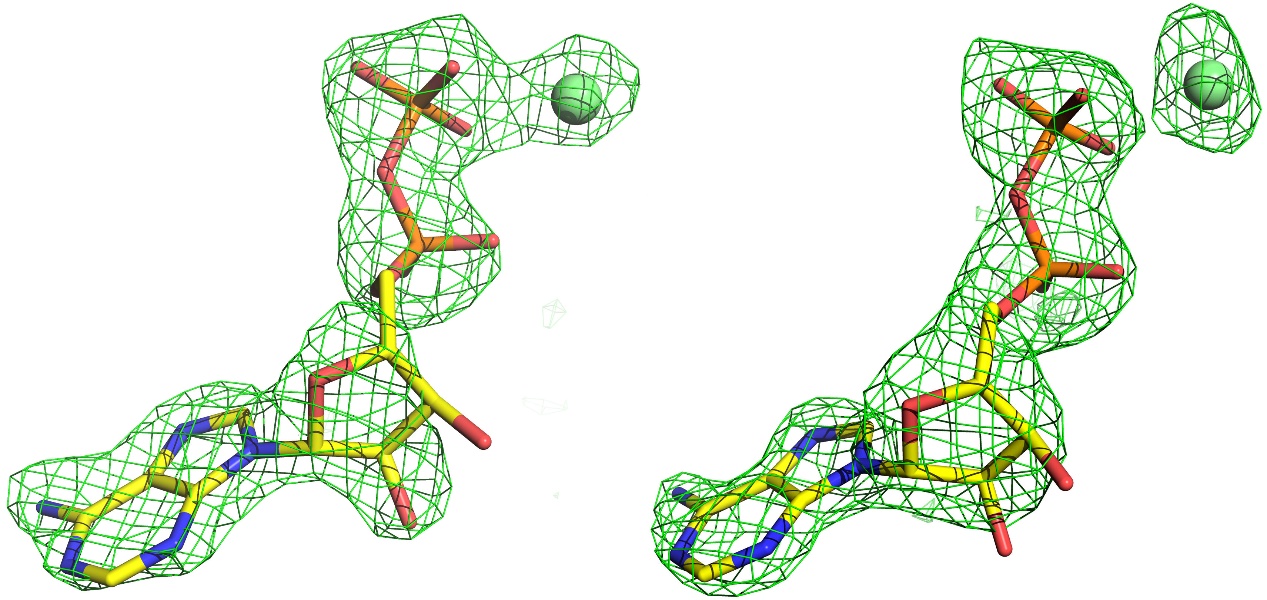
**

**Figure S2.** **Omit maps of bound nucleotides.**

The Fo-Fc electron density (green) contoured to 3 σ showing evidence for bound Mg^2+^ ion and ADP in chain A (left) and chain B (right).

**
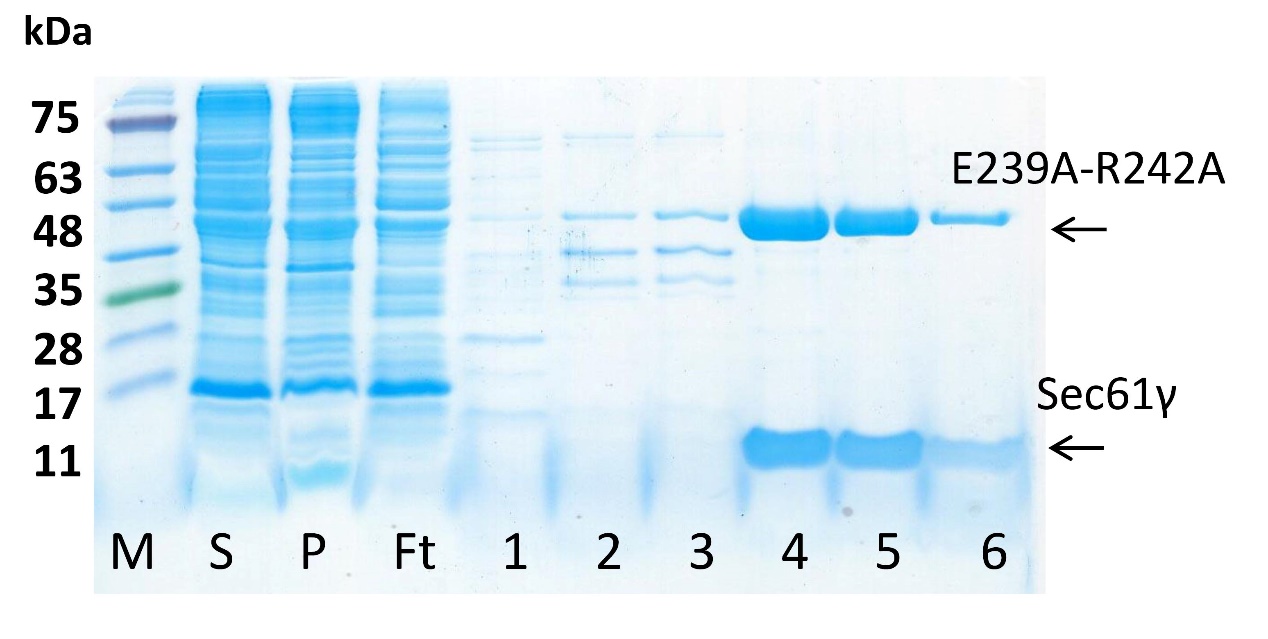
**

**Figure S3. SDS-PAGE analysis of pulldown assay.** The Pt-Get3a mutant E239A_R242A was purified using the C-terminal 6×His-tag-fused Sec61γ TA substrate by examining recombinant coexpression. M, protein marker; S, supernatant; P, pellet; Ft, flow through. We used ~10-12% Tris-tricine gel for SDS-PAGE analysis.


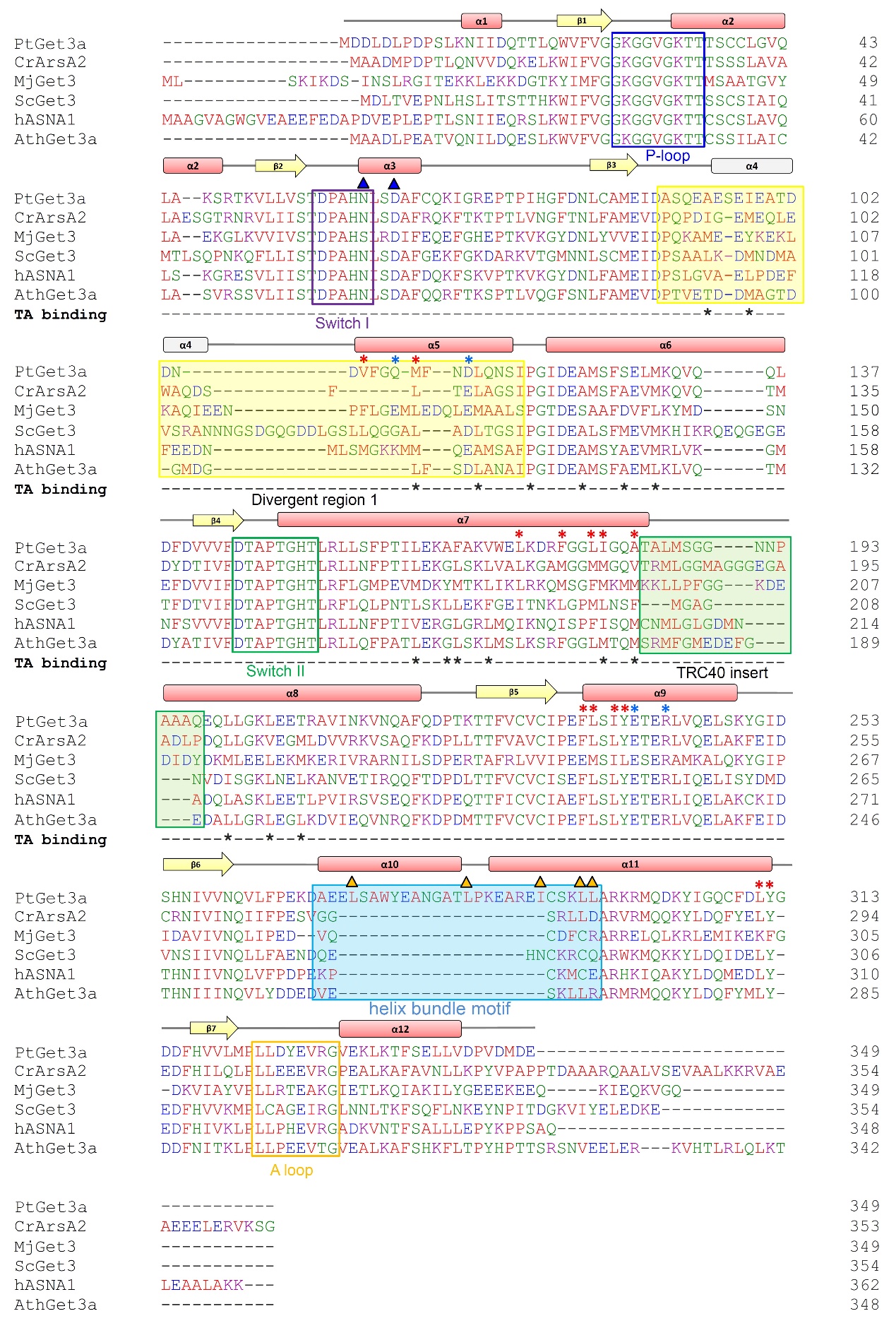


**Figure S4. Multiple sequence alignment of Get3 homologs.** Secondary structure elements (red bar for α-helix and yellow arrow for β-strand) are presented on the basis of solved crystal structures. White bar for helix α4 indicates the presence of a disordered region in Pt-Get3a. Four conserved domains of ATP-binding, namely the P- and A-loop and Switch I and II, are labeled and indicated as rectangles. The divergent region, TRC40-insert, and helix bundle motif are highlighted in yellow, green, and light blue boxes, respectively. Certain hydrophobic residues forming stable hydrophobic and hydrophilic interactions in the extensive dimer interface of A/B (or C/D) in the Pt-Get3a crystal structure are labeled with red and blue asterisks, respectively. Residues located in the dimeric four-helix bundle for the formation of an ellipsoid-shaped tetramer are represented by orange triangles. Residues involved in TA protein binding in yeast Get3 are represented as black asterisks (PDB ID: 2WOJ and 4XTR). Conserved residues that form interchain hydrogen bonds in closed *Sc*Get3 (PDB ID: 2WOJ) or *Mj*Get3 (PDB ID: 3UG6) structures to stabilize the dimer interface into a compact dimeric conformation are represented by dark blue triangles. Amino acid residues are numbered at the right side of the alignment. Pt, *Phaeodactylum tricornutum*; Cr, *Chlamydomonas reinhardtii*; Mj, *Methanocaldococcus jannaschii*; Sc, *Saccharomyces cerevisiae*; h, *Homo sapiens*; Ath, *Arabidopsis thaliana.*

**
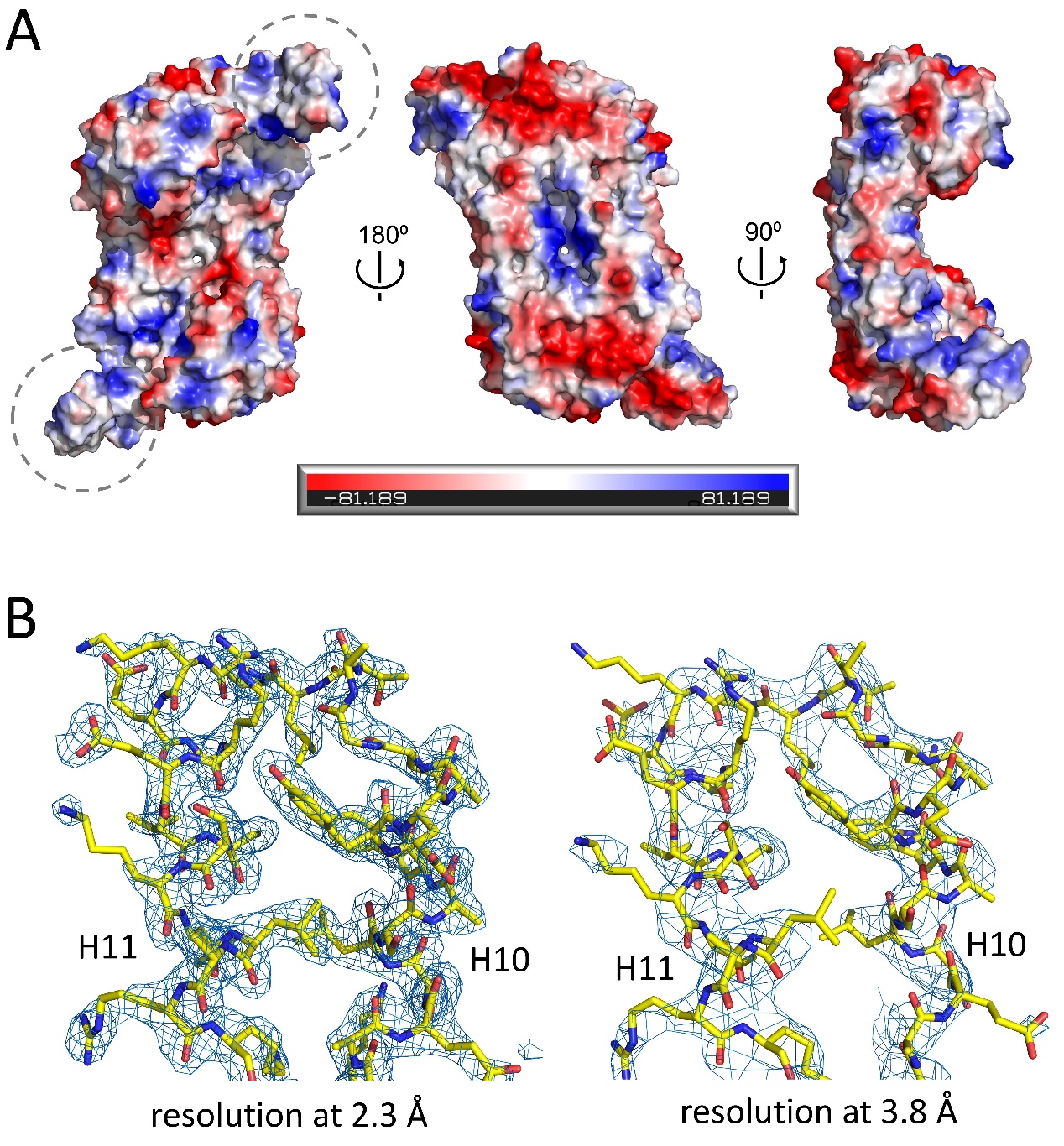
**

**Figure S5.** **Structure of the helix bundle motif.**

**A.** Three views of the electric surface potential of the parallelogram-shaped Pt-Get3 dimer. Positive charge residues are colored in blue, negative charged residues in red, and hydrophobic in white. Gray dashed circles indicate the location of the helix bundle motif. **B.** The 2Fo-Fc maps for the helix bundle motif of nucleotide-bound (left) and apo form (right) Pt-Get3a are contoured at the 1.0σ level and shown as blue mesh.

**
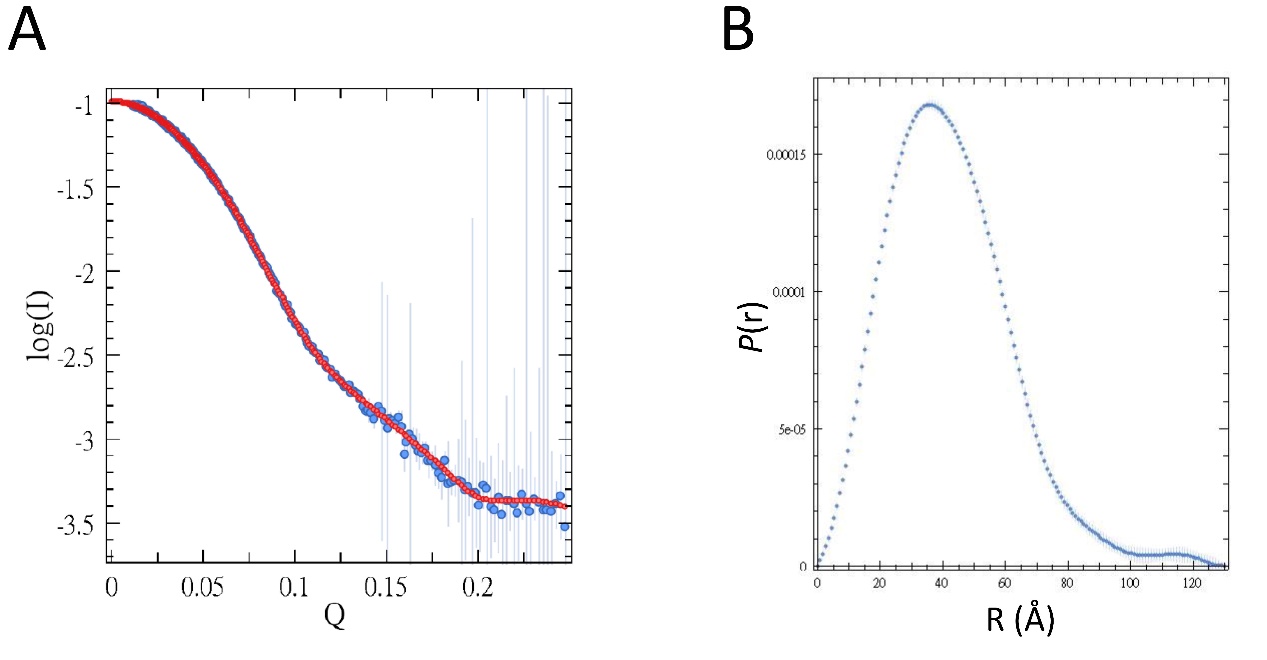
**

**Figure S6. SAXS solution structural analysis of Pt-Get3a dimer.**

**A.** The experimental scattering profile is shown as the blue circle, and the red line indicates the fitting curve for particle distance distribution function by using the GNOM program. **B.** Distance distribution *P*(*r*) representation of SAXS data from (A) using a *D*_max_ of 130 Å.

**
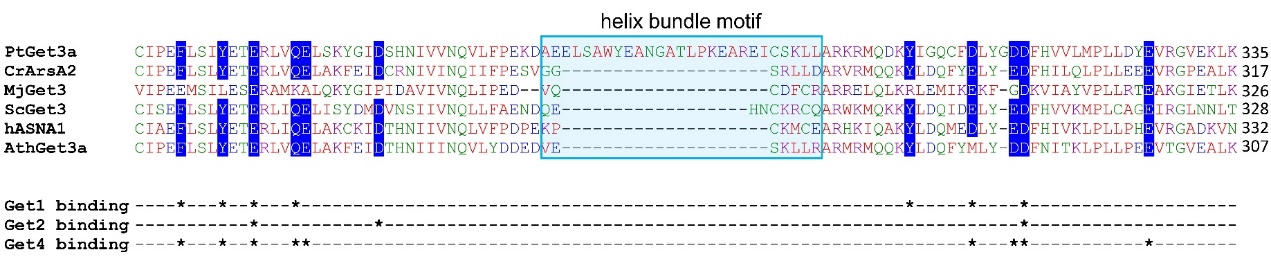
**

**Figure S7.** **Conserved residues in yeast *Sc*Get3 involved in Get1, Get2, and Get4 binding are highlighted in dark blue.** The helix bundle motif is highlighted in a light blue box. The amino acid residues are numbered at the right side of the alignment.

**
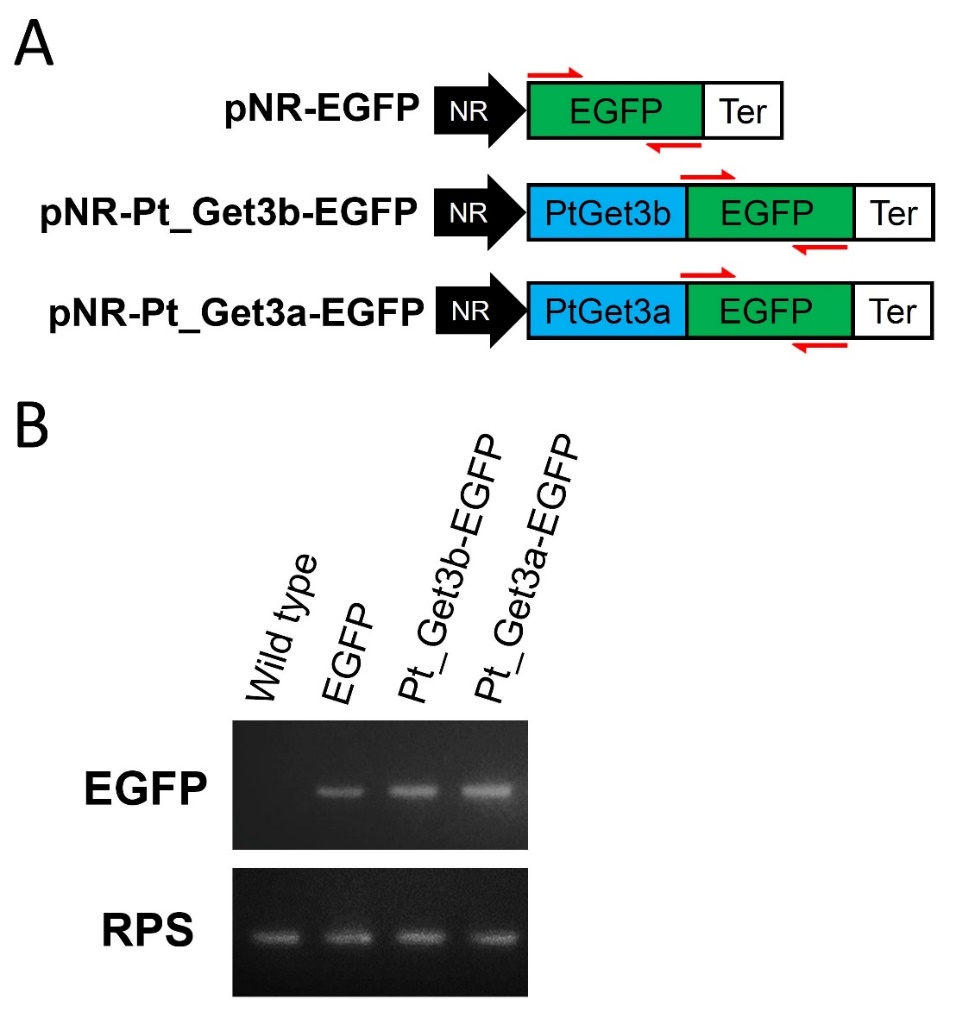
**

**Figure S8.** **Conformation of introduced fragments in transgenic diatom strains. A.** Schematic overview of the genetic composition of the exogenous gene expression system in the transgenic diatom used in this study. **B.** Genomic DNA from the wild-type and six transgenic diatom strains were isolated and analyzed through PCR. Specific primers for EGFP and ribosomal protein large subunit 30S (RPS; positive control) were used in each PCR to verify that the exogenous gene was integrated into the chromosome of *P. tricornutum*.


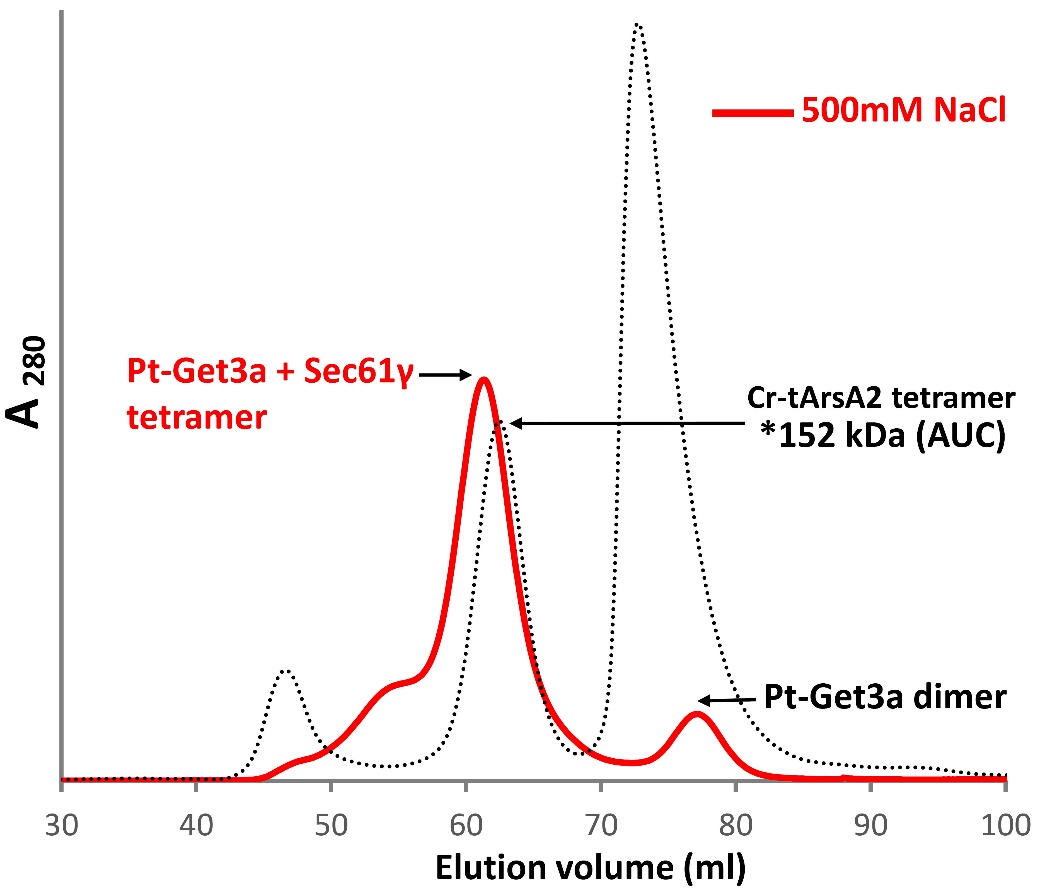


**Figure S9. SEC analysis of the Pt-Get3a/TA complex in the presence of approximately 100 mM NaCl.** The red line indicates the Pt-Get3a/Sec61γ complex in the tetrameric state as described in Figure 5C. The black dotted line indicates a protein marker with a molecular weight of 152 kDa for tetrameric Cr-ArsA2 protein.

**
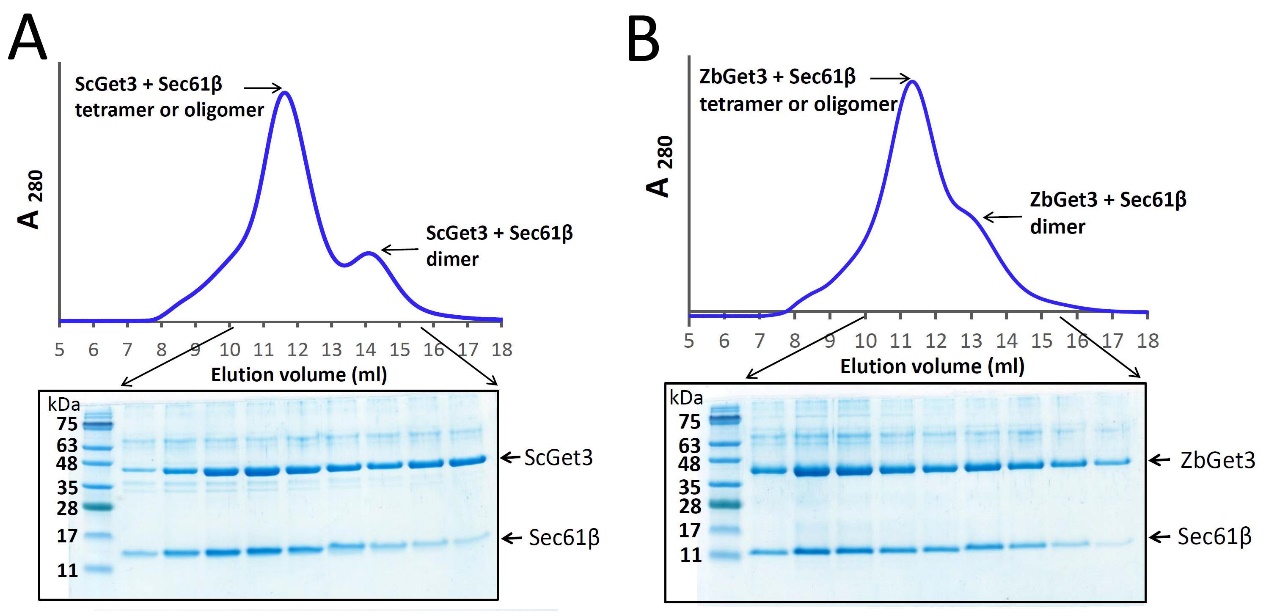
**

**Figure S10.** **SEC and SDS-PAGE analysis of Get3/TA complex.**

**A.** Sc-Get3/human-Sec61β complex. **B.** Zb-Get3/human-Sec61β complex.

**
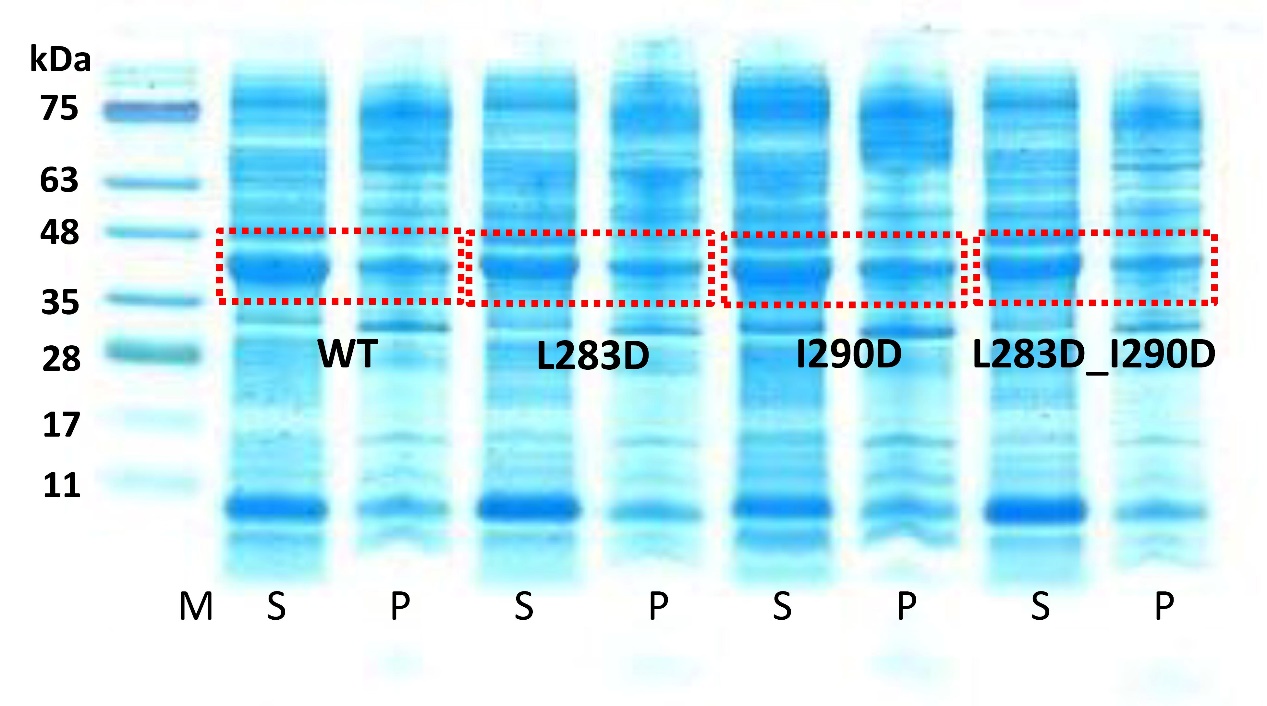
**

**Figure S11.** **SDS PAGE analysis of co-expression of Pt-Get3a and Sec61γ substrate.** Red dashed rectangles indicate the protein expression of wild type Pt-Get3a and mutants.


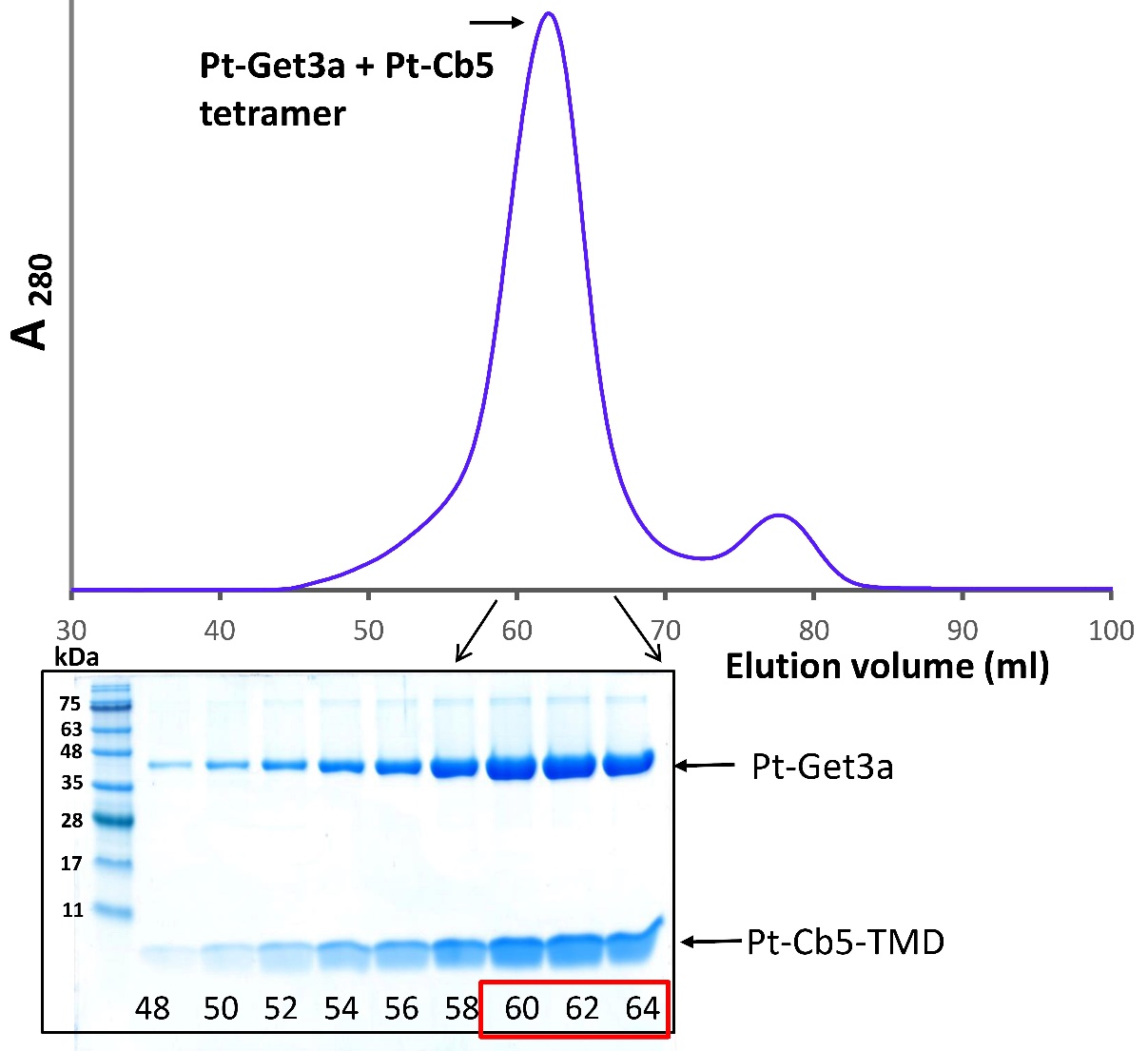


**Figure S12. TA-bound Pt-Get3a complex in the tetrameric state.**

Pt-Get3a/Cb5-TMD complex obtained through gel filtration. The analysis of approximately 62 mL of the eluted volume revealed a single sharp peak corresponding to a tetramer in solution. The red line box indicates that these elution fractions were collected for SAXS and EM analysis.

**
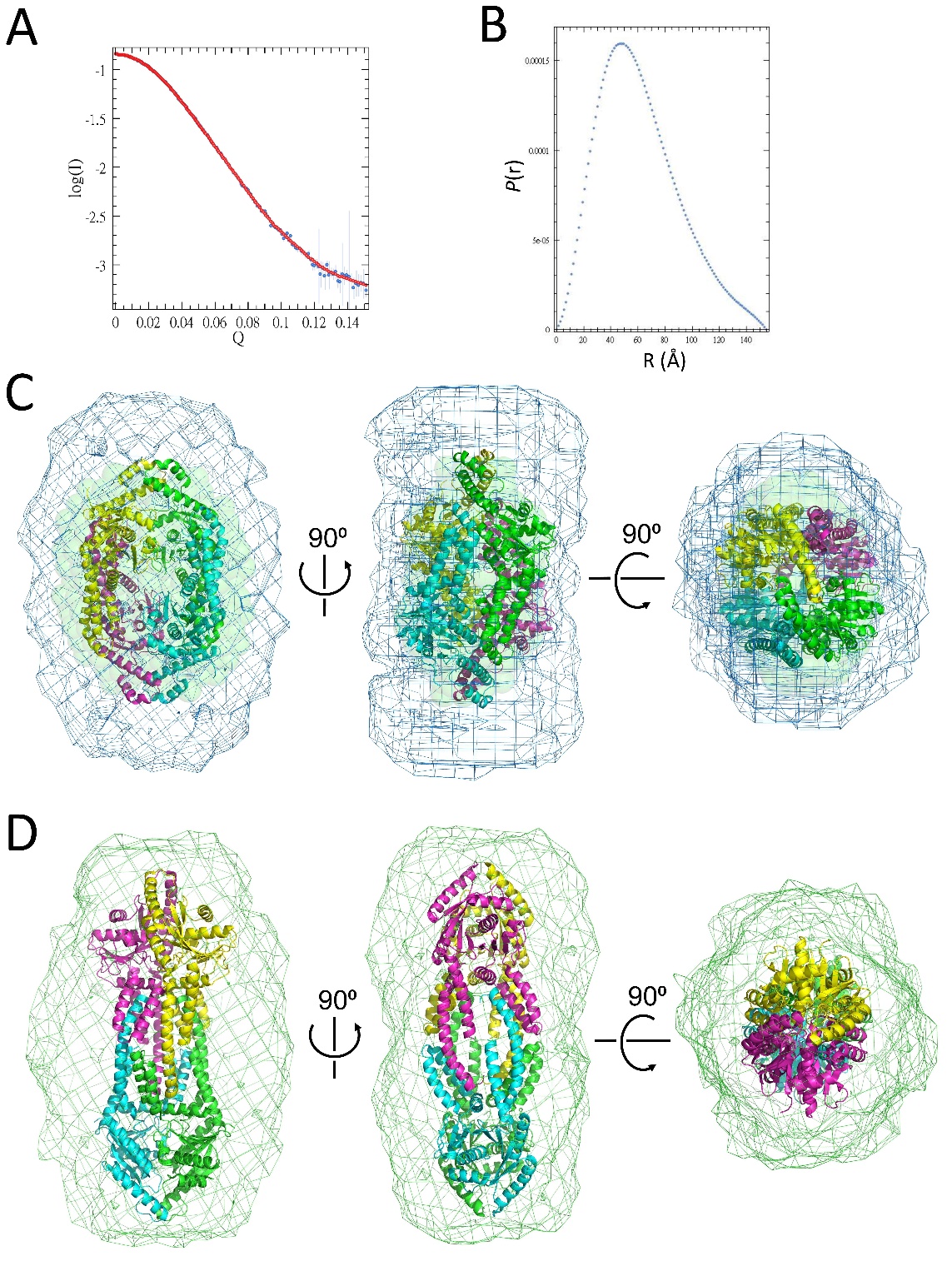
**

**Figure S13.** **SAXS solution structural analysis of the Pt-Get3a/TA tetramer complex.**

**A.** Raw SAXS scattering data were corrected for background scattering by the solvent and then extrapolated to zero concentrations by using the PRIMUS program. **B.** Distance distribution *P*(*r*) representation of SAXS data (a) at a *D*_max_ of 155 Å. **C.** SAXS structure of the Pt-Get3a/Pt-Cb5 TMD tetramer complex in solution. The averaged molecular SAXS envelope is shown in cyan mesh superimposed on the ellipsoid-shaped Pt-Get3a tetramer of the crystal structure with the same color code as shown in Figure 2A. The filtered envelope is shown in semitransparent green spheres by DAMFILT, which removes low occupancy and poorly connected atoms from the averaged envelope. **D.** Similar to **(C)**, but superimposed on the dumbbell-shaped tetramer structure of *Mj*Get3 (PDB ID: 3UG6) (green mesh).

**
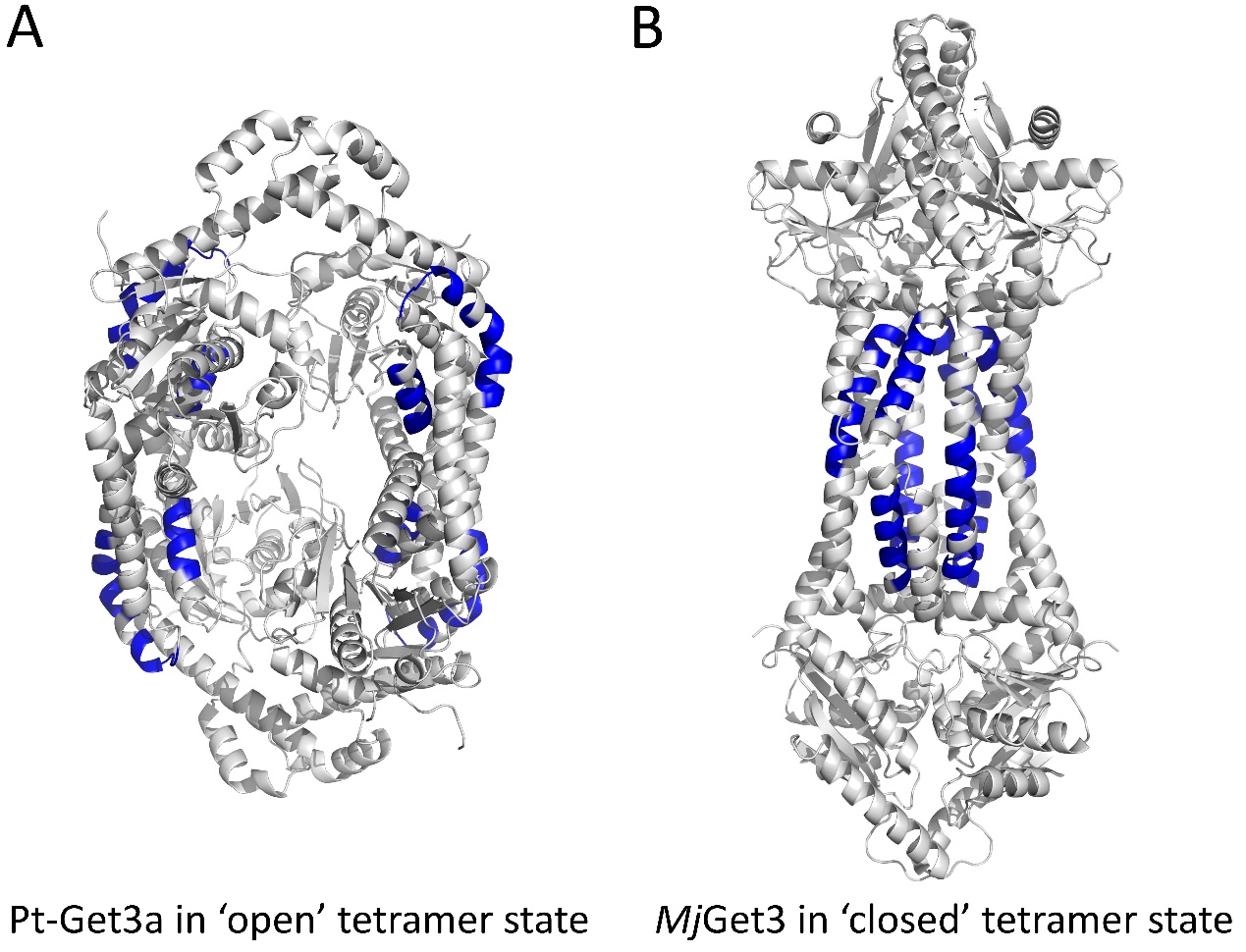
**

**Figure S14.** **The α-helical subdomain of the open Pt-Get3a tetramer and closed *Mj*Get3 tetramer.**

**A.** The dark blue ribbons indicate that regions located at the top of the α-helical subdomain may play an essential role in mitochondria-TA substrate (Cr-TOM5) binding. **B.** The equivalent regions, helices α5, and α7-α8 are colored in dark blue in *Mj*Get3 (PDB ID: 3UG6).

**
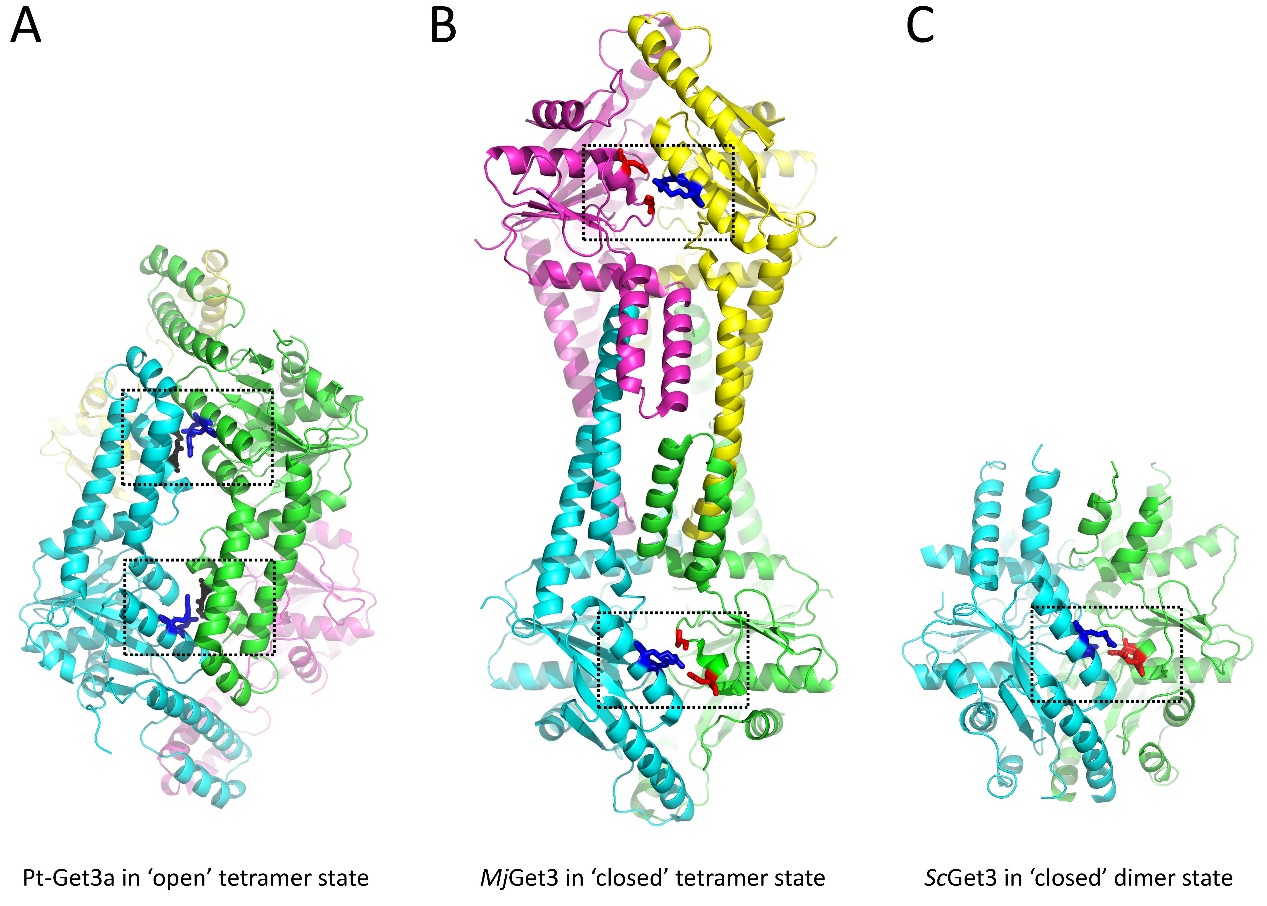
**

**Figure S15.** **Structural comparison.**

**A.** In the Pt-Get3a tetrameric structure, the two strictly conserved residues Glu239 and Arg242 (dark blue) of helix α9 form interchain salt bridges with the conserved Asp113 (black) of helix α5. **B.** The equivalent residues, Glu253 and Arg256 (dark blue) in *Mj*Get3 form interchain hydrogen bonds with other two conserved residues Ser67 and Asp70 (red) in the closed form structure (*Mj*Get3 PDB ID: 3UG6). **C.** The equivalent residues, Glu251 and Arg254 (dark blue) in yeast *Sc*Get3 form interchain hydrogen bonds with other two conserved residues Asn61 and Asp64 (red) in the closed form structures (*Sc*Get3 PDB ID: 2WOJ). The conserved residues are highlighted in black dotted boxes.
